# Glucocorticoids mediate induction of microRNA-708 to suppress ovarian cancer metastasis through targeting Rap1B

**DOI:** 10.1038/ncomms6917

**Published:** 2015-01-08

**Authors:** Kai-Ti Lin, Yu-Ming Yeh, Chi-Mu Chuang, Scarlett Y. Yang, Jer-Wei Chang, Shu-Pin Sun, Yi-Shiang Wang, Kuan-Chong Chao, Lu-Hai Wang

**Affiliations:** 1 Institute of Molecular and Genomic Medicine, National Health Research Institutes, 35 Keyan Road, Zhunan, Miaoli Country 350, Taiwan; 2 Department of Obstetrics and Gynecology, Taipei Veterans General Hospital, No. 201, Sector 2, Shipai Road, Beitou District, Taipei City 11217, Taiwan; 3 Institute of Clinical Medicine, School of Medicine, National Yang-Ming University, Taipei 11221, Taiwan; 4 Institute of Molecular Medicine, National Tsing-Hua University, Hsinchu 30013, Taiwan

## Abstract

Glucocorticoids are widely used in conjunction with chemotherapy for ovarian cancer to prevent hypersensitivity reactions. Here we reveal a novel role for glucocorticoids in the inhibition of ovarian cancer metastasis. Glucocorticoid treatments induce the expression of miR-708, leading to the suppression of Rap1B, which result in the reduction of integrin-mediated focal adhesion formation, inhibition of ovarian cancer cell migration/invasion and impaired abdominal metastasis in an orthotopic xenograft mouse model. Restoring Rap1B expression reverts glucocorticoid-miR-708 cascade-mediated suppression of ovarian cancer cell invasion and metastasis. Clinically, low miR-708 and high Rap1B are found in late-state ovarian tumours, as compared with normal, and patients with high miR-708 show significantly better survival. Overall, our findings reveal an opportunity for glucocorticoids and their downstream mediators, miR-708 or Rap1B, as therapeutic modalities against metastatic ovarian epithelial cancer.

## Abstract (web summary)

Glucocorticoids show promise for the treatment of ovarian cancer. Here the authors show that glucocorticoids transcriptionally induce the tumour suppressor miR-708, which is downregulated in ovarian cancer, especially in late stages and metastatic tumours.

## Graphical abstract

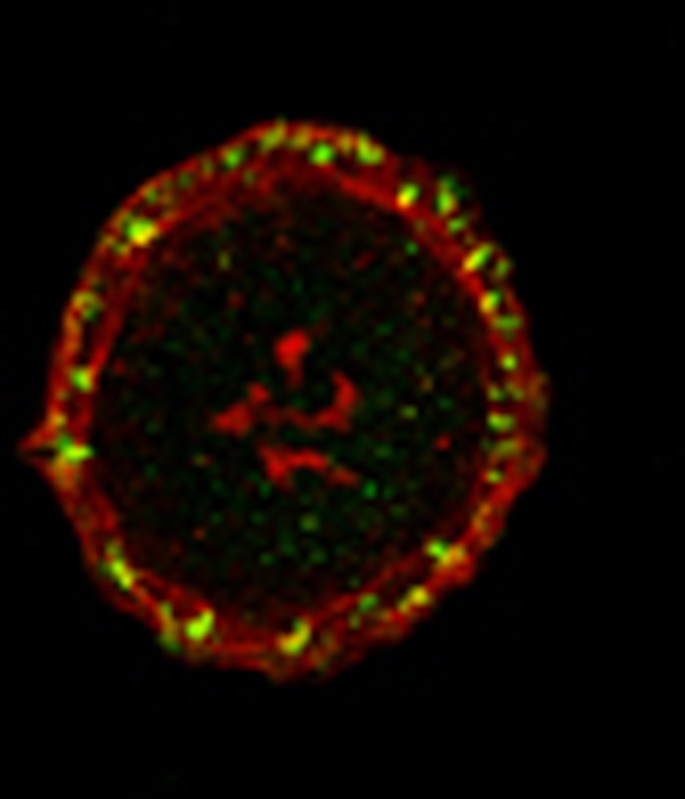

Ovarian cancer is the sixth most common cancer in women. Approximately 225,500 women worldwide are diagnosed with this disease annually, with estimated 140,200 associated deaths^1^. Most patients already harbour metastasis upon initial diagnosis, and prognosis is poor with current standard therapies^2^,^3^. Studies that devise novel therapeutic strategies against ovarian cancer metastasis are urgently warranted to improve patient outcomes.

Glucocorticoids (GCs) are known to exert pronounced effects on metabolism, differentiation, proliferation and survival of cells. Synthetic GCs, such as dexamethasone (DEX), are widely used as a pre-medication during chemotherapy to prevent hypersensitivity reactions in many cancer types^4^. Some *in vitro* studies suggest that GCs inhibit chemotherapy-induced ovarian cancer cell apoptosis^5^,^6^,^7^, raising the efficacy concerns. However, when comparing ovarian cancer patients who received chemotherapy concurrently with GCs and those without, there was no significant difference in survival^8^. Nevertheless, other than the use of GCs at the period of chemotherapy, currently no comprehensive studies suggest the other treatment possibilities of GCs, which might be beneficial to inhibit cancer metastasis as suggested by our current study. In terms of cell migration/invasion, GCs seem to play suppressive roles through a number of different mechanisms, such as downregulation of RhoA^9^, Matrix Metalloproteinase 2/9 (MMP2/9), interleukin-6 and vascular endothelial growth factor expressions^10^, or by induction of E-Cadherin^11^. To date, no prospective analysis has assessed the effect of synthetic GCs on the tumour growth or metastasis of ovarian cancers. More studies are needed to clarify the exact roles and optimal time of GC administration in ovarian cancer therapy.

MicroRNA (miRNA)-708 has been reported to suppress cancer progressions in certain types of cancers^12^,^13^,^14^. Studies so far showed that miR-708 inhibited tumour growth either through targeting anti-apoptotic pathways^12^ or by depleting CD44^+^ cells^13^. Expression of miR-708 impaired breast cancer metastasis via modulation of neuronatin^14^. Despite these advances recognizing miR-708 as a potential tumour suppressor, its role in ovarian cancer has not been reported and the detailed mechanism of how miR-708 is under regulation remains elusive. In addition, it is likely that there exist additional miR-708 targets involved in the regulation of ovarian cancer progression and metastasis.

Synthetic GC treatments are widely used to support chemotherapy. However, little is known about the regulation of miRNAs through the GC-mediated signalling pathways. In the present study, we report the finding that GC treatments play a suppressive role in ovarian cancer metastasis via the induction of miR-708. Expression of miR-708 results in decreased ovarian cancer cell migration/invasion and metastasis mainly through targeting Rap1B. Furthermore, clinical data reveal that low miR-708 and high Rap1B levels are associated with advanced-stage ovarian cancer, and high miR-708 predicts significantly better survival. Overall, our study reveals a potential role and underlying mechanism of GCs in the suppression of ovarian cancer metastasis.

## Results

### MiRNA-708 is downregulated in metastatic ovarian cancer

**Figure 1:**
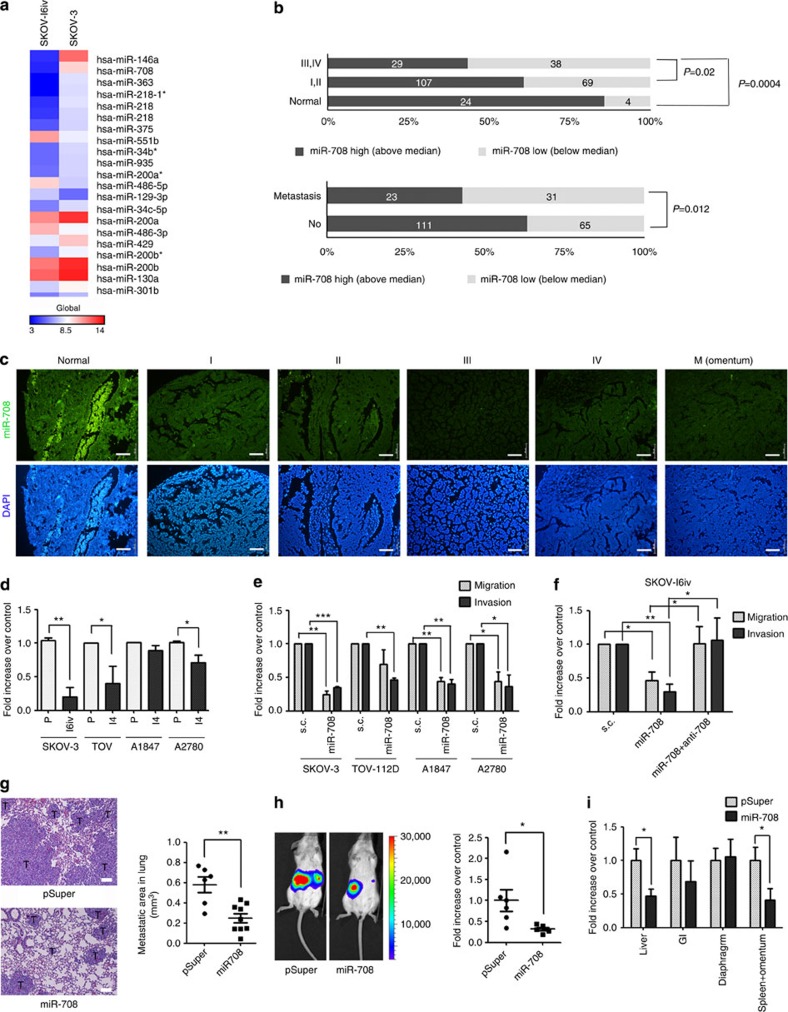
MiR-708 acts as ovarian cancer metastasis suppressor. (**a**) Heatmap of 21 most differentially regulated miRNAs from miRNA array of SKOV (Right) and SKOV-I6iv (Left). (**b**,**c**) Expression of miR-708 in ovarian tumours by FISH analysis of commercial tissue arrays. Samples were subgrouped by tumor stages or whether metastasis occurred. The statistical significance was determined using *χ*^2^ test (**b**). The representative images from different stages were shown in **c**. Scale bars: 20 μM. (**d**) Real-time RT-PCR analysis of miRNA-708 levels in SKOV-3, TOV-112D, A1847, A2780, and their derived representative invasive lines. (**e**) Ovarian cancer cells were transfected with precursor miR-708 or scrambled control (s.c.). Twenty-four hours later, cells were subjected to migration (16 h) and invasion (24 h) assays. (**f**) SKOV-I6iv cells transfected with miR-708, miR-708 together with anti-miR-708 or s.c. were incubated for migration (6 h) and invasion (8 h) assays. (**g**) Left: histology of metastasized lungs from mice intravenously injected with SKOV-I6iv cells stably expressing miR-708 (*n*=9; bottom) or vector control (*n*=6; up). Images show H&E staining. Scale bars: 100 μM. Right: statistical analysis of total metastatic area in the lung. Ten pictures were taken for each mouse. The dots represent the means of individual mice. Student’s *t*-test (two-tailed) was used to compare two groups. (**h**) Left: SKOV-I6iv cells with miR-708 expression or vector control were orthotopically injected into the bursa of the mouse ovary. The kinetics of cancer metastasis to the abdomen cavity was monitored by BLI. Representative BLIs are shown at day 28 after implantation. Right: quantification results of abdominal metastases by BLI measurements (Total BLI minus primary tumour BLI). Data are normalized to the mean of vector control group (*n*=6). Student’s *t*-test (two-tailed) was used to compare the two groups. (**i**) Individual abdominal organ metastasis was measured 30 days after orthotopic implantation using real-time RT–PCR. Human-specific GAPDH levels were used to quantify metastatic SKOV-I6iv human cells. Data represent normalized means±s.e.m. (*n*=10; data were combined from two separate experiments). Data in **d**–**f** represent normalized means±s.d. (*n*=3). Student’s *t*-test (two-tailed) was used for statistical analysis (*P<0.05; **P<0.01; ***P<0.001).

To elucidate the differential expression of miRNAs during ovarian epithelial cancer metastasis, the *in vivo* selected lung metastasized the SKOV-3 cell line (SKOV-I6iv)^15^, and its parental line, SKOV-3, were subjected to miRNA array analysis. The heatmap demonstrated differentially expressed miRNAs among SKOV-3 and SKOV-I6iv cells (Fig. 1a, Supplementary Table 2). MiRNA-708 presented as the second most downregulated miRNA in the SKOV-I6iv cells compared with parental SKOV-3 cells. Several previously reported metastasis-related miRNAs were also identified in our array results, including miR-146a (ref. 16), miR-200s (ref. 17) and miR-218 (ref. 18), validating the data reliability. We then analysed the expression of miR-708 in 271 human ovarian cancer specimens from commercial tissue arrays. The miR-708 level in primary tumours was significantly decreased in patients with advanced stages (III/IV) or harbouring metastasis, as compared with the normal ovary tissue or early-stage tumours (I/II; Fig. 1b,c). Real-time reverse transcriptase (RT)–PCR showed a significant suppression of miR-708 levels in SKOV-I6iv cells (Fig. 1d), as well as in two of the three *in vitro*-selected invasive ovarian cancer cell lines, TOV-112D and A2780 (ref. 15). Although suppression of miR-708 in the A1847- and A2780-derived invasive cell lines (A1847-I4 and A2780-I4) was not as significant as in the SKOV-3 or TOV-112D cells, depletion of endogenous miR-708 by anti-miR-708 resulted in increased migration and invasion abilities in A1847, A2780 and their derived invasive cell lines (Supplementary Fig. 1a). Cell migration assays were performed by transwell Boyden chambers and also by scratch wound-healing assays. Cell invasion assays were performed by transwell Boyden chambers pre-coated with matrigel. The Matrigel used for coating invasion chambers resembles the extracellular matrix, and this *in vitro* assay is widely used to mimic invasion through the basement membrane by metastatic cancer cells. The role of miR-708 in ovarian cancer cell migration/invasion was then examined. Expression of miR-708 resulted in a significant inhibition of cell migration/invasion in transwell assay among all ovarian cancer lines tested (Fig. 1e), as well as inhibition in wound-healing migration assay (Supplementary Fig.1b). The SKOV-I6iv cells displayed higher migration/invasion ability, as compared with SKOV-3 cells, and this ability was inhibited by miR-708 (Supplementary Fig. 1c). Moreover, we found that anti-miR-708, an antagonist of miR-708, fully reversed the inhibition of migration/invasion by miR-708 in SKOV-I6iv cells (Fig. 1f).

The suppression of ovarian cancer metastasis by miR-708 in mouse models was examined. SKOV-I6iv cells stably expressing miR-708 (Supplementary Fig. 1d), with significantly reduced migration/invasion ability (Supplementary Fig. 1e), were injected into the tail vein of severe combined immunodeficient (SCID) mice. Mice in the miR-708-expressing group demonstrated significantly reduced lung metastatic tumour areas relative to the control (Fig. 1g). No metastases were observed from organs other than the lung in this model. SKOV-I6iv cells expressing miR-708 were also orthotopically injected into mouse ovarian bursa, and the metastasis status was monitored by bioluminescence imaging (BLI) facilitated by stable expression of the *luciferase* gene. BLI analysis indicated that expression of miR-708 resulted in significantly reduced abdominal metastasis relative to the control (Fig. 1h, Supplementary Fig. 1g). Individual organ metastases were determined by the operator’s observation (Supplementary Fig. 1h), or by quantification of human-specific GAPDH RNA levels (Fig. 1i). Impairment of metastasis was observed in the miR-708 expression group among various abdominal organs, including the liver, omentum and gastrointestinal (GI) tract, as compared with the control. Notably, the tumour sizes were significantly reduced in miR-708-expressing tumors (Supplementary Fig.1i), while cell proliferation rates *in vitro* were not affected (Supplementary Fig.1j). Overall, our results demonstrate the ability of miR-708, which is downregulated in the advanced tumour types, to attenuate ovarian cancer-cell migration/invasion and metastasis.

### GC signalling controls miRNA-708 transcription

**Figure 2:**
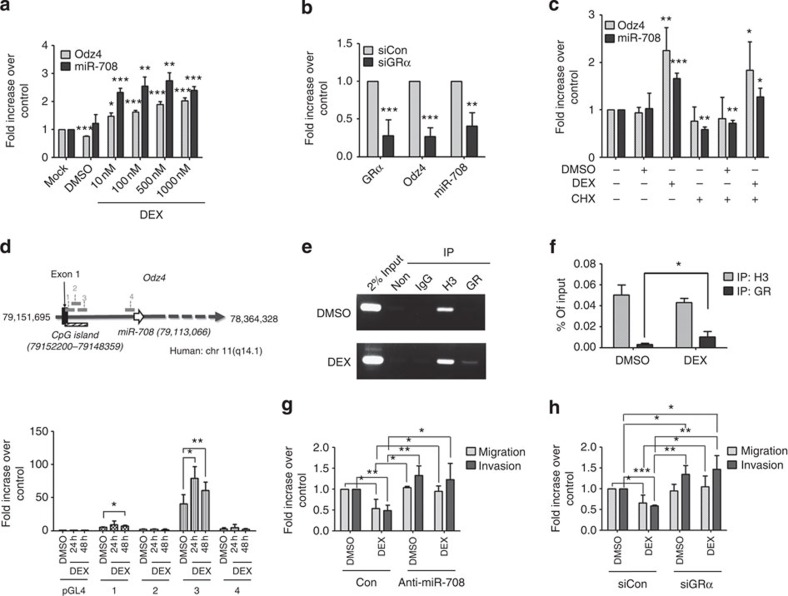
Glucocorticoid signalling induces miR-708 and *ODZ4* transcription. (**a**) Expression of *ODZ4* and miR-708 in SKOV-3 cells 72 h after treatments with increasing amount of DEX (10–1,000 nM). (**b**) Expression of *GRα*, *ODZ4* and miR-708 in SKOV-3 cells transfected with siGRα or siCon, and then treated with 1 μM DEX for 72 h. (**c**) Expressions of *ODZ4* and miR-708 in SKOV-3 cells pretreated with 10 μM cycloheximide overnight followed by 1 μM DEX for 48 h. (**d**) Up: schematic graph illustrating genomic locations of *ODZ4* and miR-708 genes. The putative promoter regions tested for luciferase activities are indicated as short grey bars with assigned numbers (promoter-1 to -4). Bottom: SKOV-3 cells were transfected with pGL4, or pGL4 with promoter-1 to -4, treated with 1 μM DEX for 24 or 48 h, and assayed for luciferase activity. (**e**,**f**) SKOV-3 cells were treated with 1 μM DEX for 3 h, and the ChIP assay was performed on the promoter-3 region. The PCR product was analysed with agarose gel electrogenesis (**e**) or real-time PCR (**f**). (**g**,**h**) SKOV-3 cells transfected with anti-miR-708, control (**g**), siGRα or siCon (**h**) were treated with 1 μM DEX overnight, and then incubated for migration (8 h) and invasion (16 h) assays. DMEM/10% FBS, together wth 1 μM DEX or DMSO control, served as a chemoattractant. Data in **a**–**h** represent normalized means±s.d. (*n*=3). Student’s *t*-test (two-tailed) was used for statistical analysis (*P<0.05; **P<0.01; ***P<0.001).

A study searching for differential miRNA expression in acute lymphoblastic leukemia (ALL) samples revealed that upregulation of miR-708 was associated with *in vivo* GC response in childhood ALL^19^; therefore, GC-mediated signalling may be involved in the regulation of miR-708 expression. Since miRNA-708 is an intronic miRNA encoded in the first intron of the *ODZ4* gene (also known as Teneurin-4)^12^,^20^, we tested both *ODZ4* and miR-708 expressions in the presence of synthetic GC, DEX. Both miR-708 and *ODZ4* expressions were significantly induced by the treatment with DEX (Fig. 2a) and another synthetic GC, prednisolone (Supplementary Fig. 2a), in SKOV-3 cells. Similar induction of miR-708 and *ODZ4* by DEX was also observed in TOV-112D cells (Supplementary Fig. 2b). Depletion of GRα by small interfering RNA (siRNA) in SKOV-3 cells demonstrated inhibition of both miR-708 and *ODZ4* expressions (Fig. 2b). A protein synthesis inhibitor, cycloheximide (CHX), was then applied to inhibit *de novo* protein synthesis before treatment with DEX. In CHX-pretreated SKOV-3 cells, DEX treatment still induced miR-708 and *ODZ4* expression (Fig. 2c) indicating that *de novo* protein synthesis was not required for GRα-induced miR-708 and *ODZ4* transcriptions.

We then sought to identify the promoter region of miR-708 responsible for GC-mediated signalling. Four potential promoter regions were identified (promoter 1–4, Fig. 2d). The upstream genomic region proximal to the *ODZ4* first intron start site contains CpG islands (3,842 bps) that are highly conserved among mammals, which may serve as the promoter region for *ODZ4* and miR-708 (promoter-1 to -3). On the other hand, the region within 2,000 bps upstream of miR-708 may contain an independent promoter for miR-708 alone (promoter-4)^14^. To localize the promoter regions, we constructed reporter plasmids carrying promoter-1 to 4 (Fig. 2d) and assayed their luciferase activities. Results demonstrated that only the promoter-3 region among the CpG islands had an over 40-fold higher promoter activity in SKOV-3 cells compared with that of the vector control (Fig. 2d). This promoter activity was further enhanced by 1.5-folds in the presence of DEX. Chromatin immunoprecipitation (ChIP) assay was then performed to confirm the recruitment of GR to the promoter-3 region in the presence of DEX (Fig. 2e,f). Our findings suggest that the promoter-3 region contains the miR-708 promoter responsible for its GC-mediated regulation in ovarian cancer cells.

To assess the function mediated by GC signalling, we examined the migration/invasion in the presence of different concentrations of DEX (10 nM, 100 nM and 1 μM). Migration/invasion was significantly reduced even by treatment with the lowest concentration of DEX in SKOV-3 cells (Supplementary Fig. 2c). We further depleted the endogenous miR-708 by anti-miR-708 in SKOV-3 cells in the presence of GCs. Migration/invasion was inhibited in both DEX- (Fig. 2g, Supplementary Fig. 2d) and prednisolone- (Supplementary Fig. 2e) treated SKOV-3 cells, and this inhibition was abrogated by anti-miR-708. Knockdown of GRα significantly abolished DEX-mediated inhibition of cell migration/invasion in SKOV-3 cells (Fig. 2h), implying that DEX-mediated inhibition of cell migration/invasion was mainly through GRα. On the other hand, knockdown of *ODZ4* did not rescue cell migration/invasion from DEX-mediated inhibition (Supplementary Fig. 2f), suggesting that the host gene itself was not involved in the GC-regulated cell migration/invasion. Overall, our results demonstrate that miR-708, together with *ODZ4*, are transcriptionally regulated by the GR-mediated signalling pathways.

### MiRNA-708 suppresses migration and invasion via Rap1B

**Figure 3:**
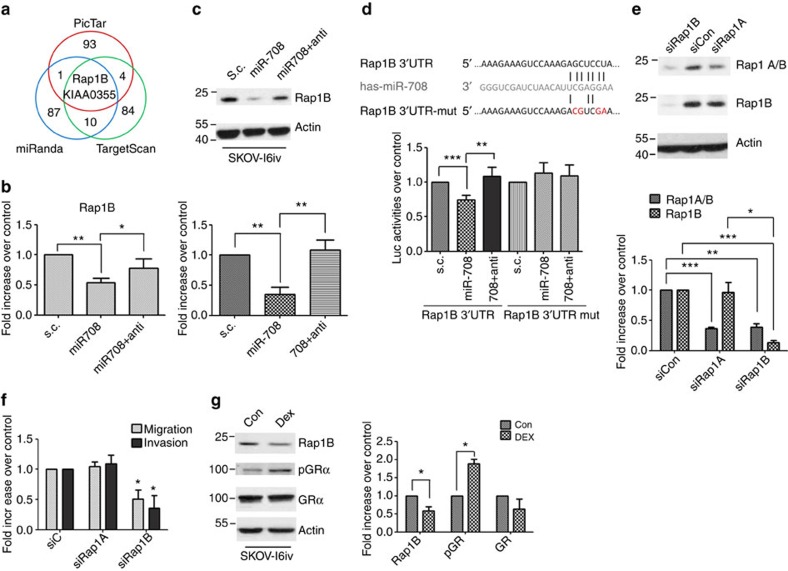
MiR-708 inhibits migration/invasion by targeting Rap1B. (**a**) Venn diagram showing the overlap of top 100 genes potentially targeted by miR-708 as predicted by the following three algorithms: TargetScan, miRanda and PicTar. (**b**) Expression of Rap1B mRNA in SKOV-I6iv cells transfected with miR-708, miR-708 plus anti-miR-708 or s.c. Data represent normalized means±s.d. (*n*=3). (**c**) Upper: expression of Rap1B proteins in SKOV-I6iv cells transfected with miR-708, miR-708 plus anti-miR-708 or s.c. Bottom: quantitative analysis of the Rap1B protein level, and normalized with the actin level. Histograms represent normalized means±s.e.m. (*n*=3). (**d**) Upper: sequence of miR-708 and the potential miR-708-binding site at Rap1B 3′ UTR. Nucleotides mutated in miR-708-binding site are shown in red. Bottom: luciferase assays demonstrating that expression of Rap1B 3′ UTR (wild-type or mutant form) by SKOV-I6iv cells transfected with miR-708, miR-708 plus anti-miR-708 or s.c. Data represent normalized means±s.d. (*n*=3). (**e**) Upper: protein expression of total Rap1 (including Rap1A and Rap1B), and Rap1B in SKOV-I6iv cells upon Rap1A, Rap1B or control siRNA transfection. Bottom: quantitative analysis of the protein level, and normalized with the actin level. Histograms represent normalized means±s.e.m. (*n*=3). (**f**) SKOV-I6iv cells transfected with Rap1A, Rap1B or control siRNA were incubated for migration (6 h) and invasion (8 h) assays. DMEM/10% FBS served as a chemoattractant. Data represent normalized means±s.d. (*n*=3). (**g**) Left: western blot analysis showing Rap1B, p-GRα and GRα expression in SKOV-I6iv cells upon treatment of 1 μM DEX for 48 h. Right: quantitative analysis of the protein level, and normalized with the actin level. Histograms represent normalized means±s.e.m. (*n*=3). Student’s *t*-test (two-tailed) was used for statistical analysis (*P<0.05; **P<0.01; ***P<0.001).

To identify miR-708-mediated downstream regulator in ovarian cancer metastasis, three target prediction algorithms (PicTar, Targetscan and miRanda) were applied. The first 100 picks from the three algorithms were selected, and from those only two genes overlapped, Rap1B and KIAA0355 (Fig. 3a). Genes predicted by more than one algorithm are listed in Supplementary Table 3. Rap1B, a known regulator of adhesion processes through integrin-mediated signalling^21^,^22^,^23^, thus stands as an excellent candidate involved in metastatic processes. Expression of miR-708 in the SKOV-I6iv cells resulted in a significant decrease of Rap1B in both mRNA (Fig. 3b) and protein (Fig. 3c), and this inhibition was abrogated upon co-expression of anti-miR-708. The Rap1B inhibition by miR-708 was also observed in other ovarian cancer cell lines (Supplementary Fig. 3a). Knockdown of the endogenous miR-708 by anti-miR-708 increased Rap1B protein levels in SKOV-3 cells (Supplementary Fig. 3b). Analysis using 3′ untranslated repeat (UTR) luciferase reporter plasmids containing the miR-708 target sequences (wild-type or mutant) on Rap1B was performed to determine whether Rap1B was a direct target of miR-708. Expression of miR-708 caused a significant decrease in luciferase activity in the wild-type Rap1B 3′ UTR, but not in the mutant form (Fig. 3d). Co-expression of anti-miR-708 fully reversed miR-708-mediated inhibition, indicating that Rap1B is a direct downstream target of miR-708. Other genes reported previously as the miR-708 direct targets^12^,^13^,^14^,^24^ were tested in SKOV-I6iv cells (Supplementary Fig. 3c). Surprisingly, other than Rap1B, only the AKT2 mRNA level was slightly reduced in the presence of miR-708. Although our data only reflected mRNA levels, and some genes might be affected translationally, we decided to focus on Rap1B as the major target of miR-708 in ovarian cancer.

Rap1B, as a member of the small GTPase Ras-proximity-1 (Rap1), shares 95% amino-acid sequence identity with Rap1A^25^. The function of Rap1B and Rap1A in ovarian cancer migration/invasion was then tested. Depletion of Rap1A or Rap1B by respective siRNAs demonstrated the expected efficiency and specificity (Fig. 3e). Migration/invasion was significantly reduced in SKOV-I6iv cells with Rap1B, but not Rap1A, knockdown (Fig. 3f, Supplementary Fig. 3d). Rap1B levels were reduced in DEX-treated SKOV-3 cells, accompanied by increased level of active GRα (Fig. 3g), connecting miR-708-Rap1B regulation to the GRα signalling cascade.

To further investigate whether Rap1B causes the differences of migration and invasion between SKOV-3 cells and its derived invasive cell line, SKOV-i6iv cells, we manipulated Rap1B expression by either overexpressing haemagglutinin (HA)-tagged Rap1B in SKOV-3 cells or siRNA depletion of Rap1B in SKOV-I6iv cells (Supplementary Fig. 3e). The overexpression of Rap1B in the less invasive SKOV-3 cells resulted in significantly increased migration/invasion, and this induction is comparable to that in the highly invasive SKOV-I6iv cells. The opposite phenomenon was also shown in SKOV-I6iv cells with Rap1B knockdown to a level comparable to that in SKOV-3 cells, resulting in a corresponding decrease in migration/invasion (Supplementary Fig. 3f). Meanwhile, we observed that the endogenous Rap1B expression was two- to three-folds higher in SKOV-I6iv cells than that in the parental SKOV-3 cells (Supplementary Fig. 3e), which is negatively correlated with endogenous miR-708 expression in these two cell lines (Fig. 1d).

### MiRNA-708 impairs integrin-mediated cell adhesion

**Figure 4:**
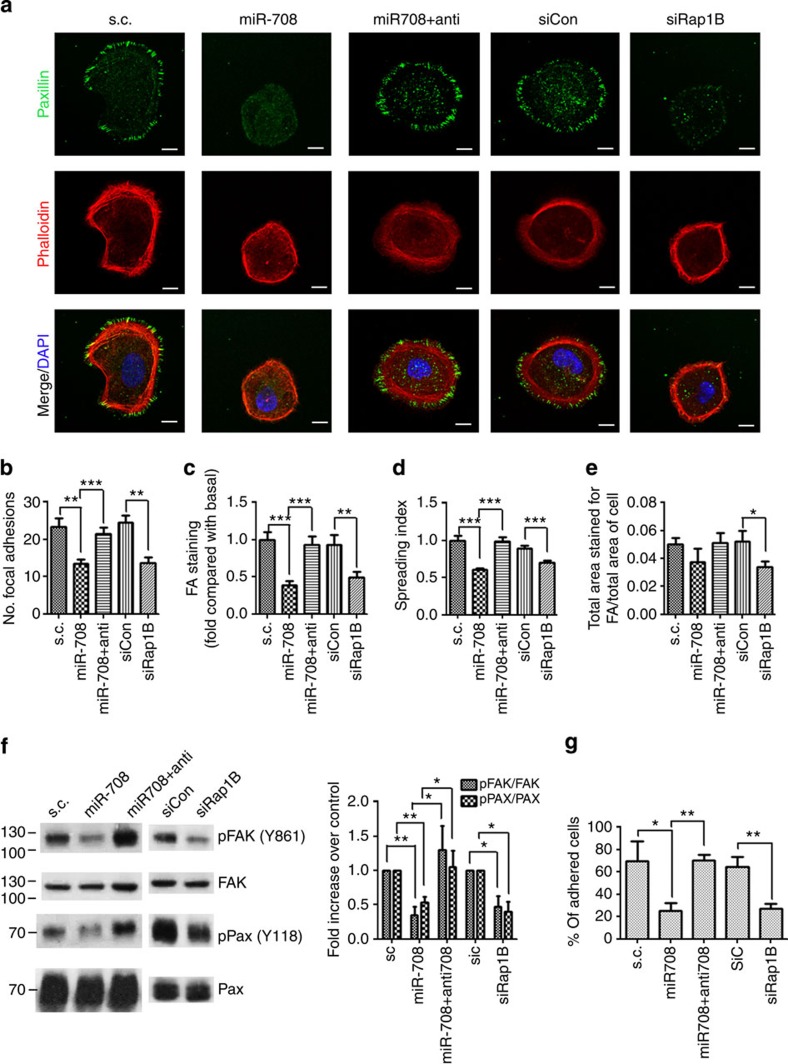
MiR-708 inhibits FA formation, cell spreading and cell adhesion. (**a**) Confocal microscopy of Paxillin (Green), β-actin (Red) and DAPI (Blue) nuclear staining, after adhering of cells to FN. SKOV-I6iv cells with different transfections were equally plated on FN-coated coverslips for 90 min. The FAs were detected. Scale bars: 10 μm. (**b**–**e**) The images were analysed to determine the number of FAs per cell (**b**), the area of the cell staining for FAs by paxillin staining (**c**) and the cell-spreading area by β-actin staining (**d**). To determine the proportion of each cell consisting of FAs, the area of FAs was divided by the total spreading area of the cell (**e**). Data in **b**–**e** represent means±s.e.m. (*n*=30). (**f**) Left: western blot analysis showing p-Paxillin, p-FAK, Paxillin and FAK expressions in SKOV-I6iv cells with different transfections. Cells were incubated on FN-coated plates for 60 min before being subjected to western blot analysis. Right: quantitative analysis of protein levels, and normalized with total FAK or paxillin levels. Histograms represent normalized means±s.e.m. (*n*=3). (**g**) Adhesion assays of SKOV-I6iv cells with GFP plus different transfections. Percentage of adhered cells was counted. Data represent means±s.d. (*n*=3). Student’s *t*-test (two-tailed) was used for statistical analysis (*P<0.05; **P<0.01; ***P<0.001).

The Rap1 signalling axis has been shown to promote invasion and metastasis through facilitating integrin-mediated actin remodelling in human pancreatic carcinoma cells^26^ and breast cancer cells^27^. The effect of miR-708 and Rap1B on the formation of focal adhesions (FAs) in SKOV-I6iv cells was examined by using immunofluorescence to monitor changes of FAs, the cytoskeleton and cell shape, following 90-min incubation on fibronectin (FN)-coated cover slips (Fig. 4a). Expression of miR-708 or knockdown of Rap1B disrupted the spreading of cells (Fig. 4d), reduced the number and intensity of FAs (Fig. 4b,c), decreased the density of FAs per spreading area of the cell (Fig. 4e), as well as decreased the recruitment of phospho-Paxillin (Y118) and phospho-focal adhesion kinase (FAK; Y861) to the FAs (Supplementary Fig. 4a,b). The inhibitory effect by miR-708 was reversed by anti-miR-708. Tyrosine 861 of FAK is the major phosphorylation site for Src^28^, and this phosphorylation is crucial for integrin signalling-mediated promotion of epithelial–mesenchymal transition and migration^29^. Western blot analysis demonstrated that miR-708 overexpression or Rap1B knockdown decreased the level of p-FAK and p-Paxillin in SKOV-I6iv cells, and this reduction was abrogated by anti-miR-708 (Fig. 4f). Meanwhile, depletion of miR-708 or expression of HA-tagged Rap1B in the parental SKOV-3 cells resulted in the increased FAs and cell-spreading abilities (Supplementary Fig. 5a–f), suggesting the possibility that miR-708 regulates formation of FAs and cell spreading via Rap1B.

We then examined the effect of miR-708 on adhesion ability. Expression of miR-708 or depletion of Rap1B significantly decreased the adhesion ability in SKOV-I6iv cells plated on FN (Fig. 4g) or Collagen-I (Supplementary Fig. 4c). These data suggest that miR-708-mediated depletion of Rap1B is sufficient to impair integrin-mediated cell adhesion through disruption of FA formation.

To further assess whether GC treatments affect formation of FAs and cell adhesion abilities, DEX was applied to stimulate GC-mediated signalling pathway in SKOV-I6iv cells. We found that DEX inhibited formation of FAs, spreading of cells, recruitment of p-Paxillin and p-FAK to FAs (Supplementary Fig. 6a–g), as well as the adhesion ability to FN-coated plates (Supplementary Fig. 6h). These data tether GCs to the miR-708-Rap1B signalling pathway in its ability to modulate cell spreading and adhesion through regulation of FA formation.

### Rap1B rescues miR-708-suppressed cell migration and invasion

**Figure 5:**
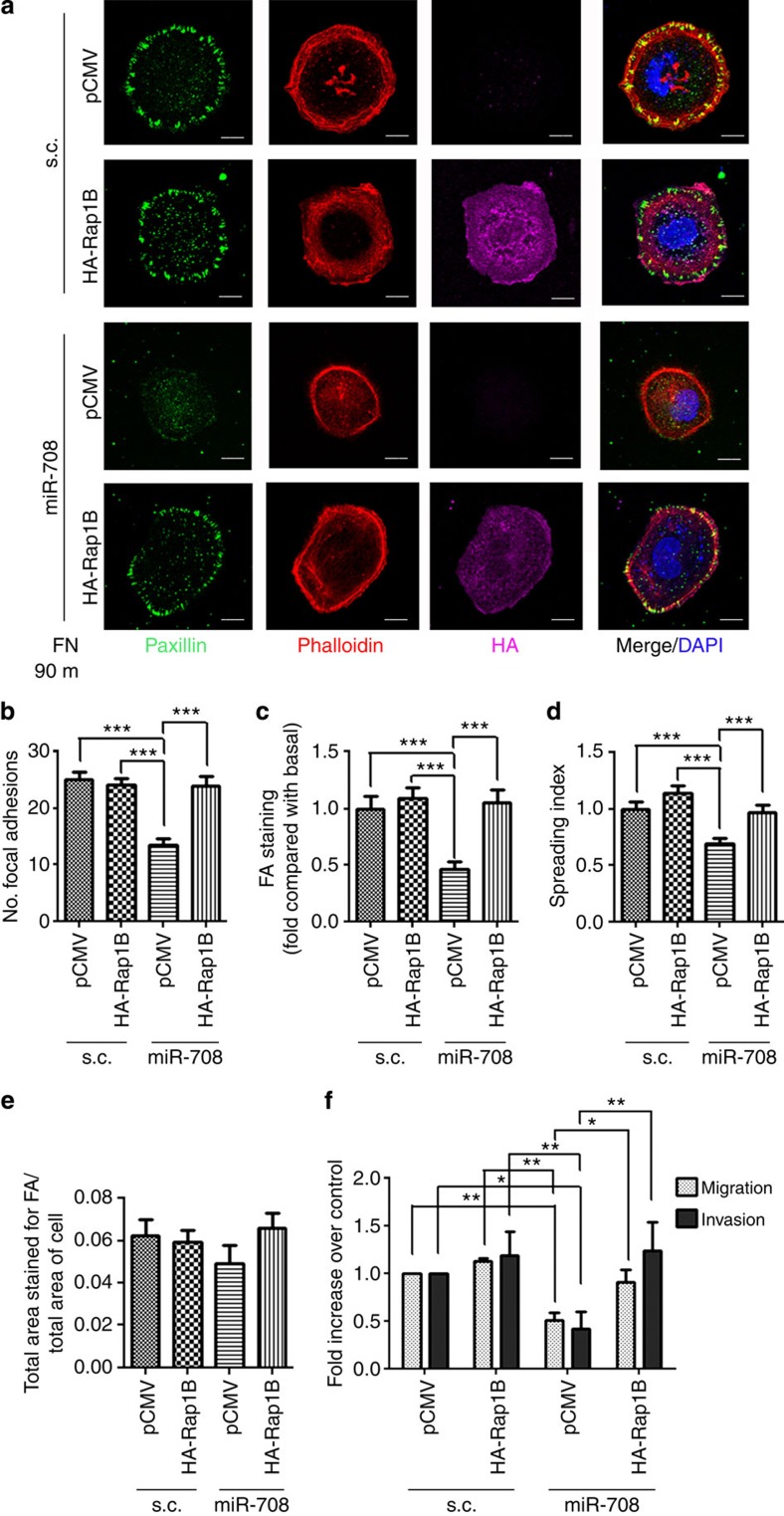
Rap1B rescues miR-708-impaired FA formation and migration/invasion. (**a**) Confocal microscopy of Paxillin (green), β-actin (red), HA-tagged-Rap1B (purple) and DAPI (blue) nuclear staining. SKOV-I6iv cells with indicated transfections were incubated on FN-coated coverslips for 90 min. Scale Bars: 10 μm. (**b**–**e**) The images were analysed to determine the number of FAs per cell (**b**), the area of the cell staining for FAs by paxillin staining (**c**) and the cell-spreading area by β-actin staining (**d**). To determine the proportion of each cell consisting of FAs, the area of FAs was divided by the total spreading area of the cell (**e**). Data in **b**–**e** represent means±s.e.m. (*n*=30). (**f**) SKOV-I6iv cells with the indicated transfection were incubated for migration (6 h) and invasion (8 h) assay. Data represent normalized means±s.d. (*n*=3). Student’s *t*-test (two-tailed) was used for statistical analysis (*P<0.05; **P<0.01; ***P<0.001).

To determine whether Rap1B is a functionally important target of miR-708 in ovarian cancer cells, rescue experiments were performed. HA-Rap1B rescued miR-708-mediated reduction in the number and intensity of FAs (Fig. 5a–c), as well as inhibition of cell spreading (Fig. 5a,d). The density of FAs per spreading area of the cell was also slightly inhibited by miR-708, and re-expression of HA-Rap1B rescued this inhibition (Fig. 5e). The impaired recruitments of p-Paxillin (Supplementary Fig. 7a) and p-FAK (Supplementary Fig. 7b) at FAs by miR-708 were also restored with HA-Rap1B expression. More importantly, the inhibition of migration/invasion by miR-708 was reversed in cells co-expressing HA-Rap1B (Fig. 5f). Apparently, additional Rap1B above the endogenous level was not needed and was not able to significantly further promote FAs, spreading or migration/invasion in SKOV-I6iv cells. Overall, our results indicate that Rap1B is functionally important for miR-708-mediated regulation of ovarian cancer cell migration/invasion.

### GC treatments suppress ovarian cancer metastasis

**Figure 6:**
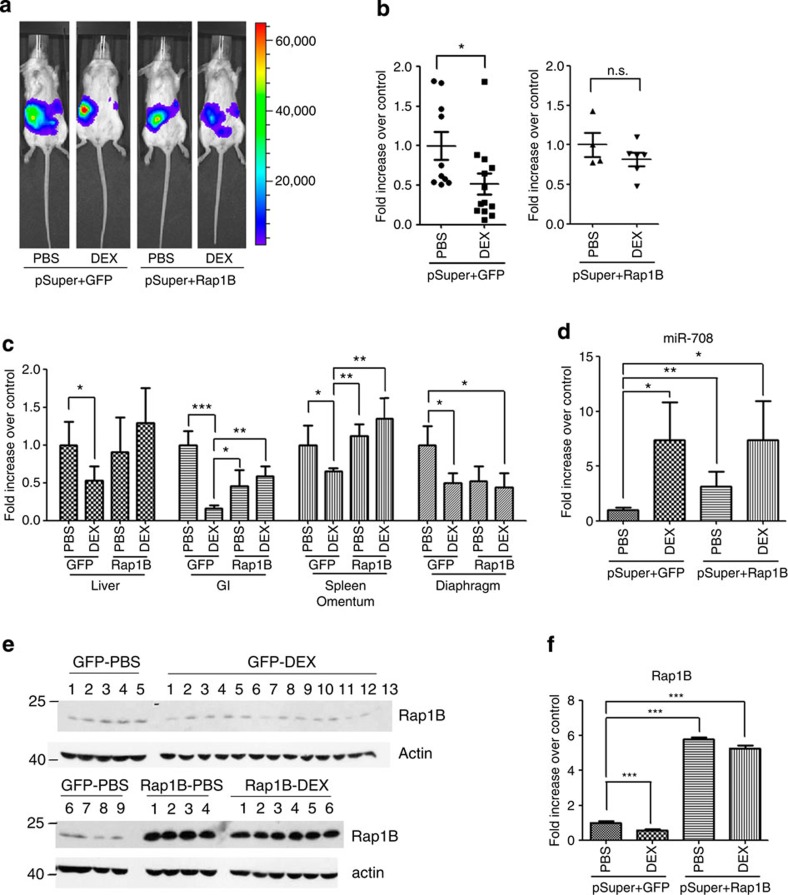
Glucocorticoids suppress ovarian cancer cell abdominal metastasis. (**a**) SKOV-I6iv cells stably expressing pSuper-GFP-Luc plus Rap1B or GFP were orthotopically injected into the mouse ovary. The kinetics of cancer abdominal metastasis was monitored by BLI. Representative BLI images are shown on day 28 after implantation. (**b**) Quantification results of abdominal metastases by BLI measurements (total BLI minus primary tumour BLI). Data are normalized to the means of PBS-GFP or PBS-Rap1B (*n*=4–13; data were combined from two separate experiments). (**c**) Individual abdominal organ metastasis was measured by human-specific GAPDH levels to quantify metastatic SKOV-I6iv cells. Data are normalized to the means of PBS–GFP from individual organ±s.e.m. (*n*=4–8). (**d**) Expression of miR-708 in primary orthotopically implanted ovarian tumors. Data are normalized to the means of PBS-GFP±s.e.m. (*n*=4–8). (**e**) Western blot analysis of Rap1B expression from orthotopically implanted primary tumours. (**f**) Quantitative analysis of the Rap1B protein level from **e**, and normalized with the actin level. Histograms represent normalized means±s.e.m. Student’s *t*-test (two-tailed) was used for statistical analysis (*P<0.05; **P<0.01; ***P<0.001; n.s., no significance).

So far, we have demonstrated that GC-mediated signalling resulted in the inhibition of ovarian cancer cell migration/invasion mainly through miR-708-Rap1B regulation. We next examined this pathway in the *in vivo* mouse model. We first confirmed that miR-708 expression was significantly induced by the treatment with DEX in SKOV-I6iv cells (Supplementary Fig. 8a), and then we established SKOV-I6iv-pSuper-GFP-Luc cells stably expressing Rap1B or green fluorescent protein (GFP) by lentiviral infection system for animal study. These two cell lines presented similar proliferation rates (Supplementary Fig. 8b), and cells were implanted orthotopically to ovarian bursa of the SCID mice. Five days after implantation, we started intraperitoneal DEX administration three times a week until day 30. BLI data indicated that DEX injections resulted in a significant reduction of abdominal metastasis in the GFP-expressing group, while not much difference was found between PBS- and DEX-treated mice with Rap1B re-expression (Fig. 6a,b; Supplementary Fig. 8d). There were no significant differences in the sizes of primary tumours between the PBS–GFP and Rap1B groups with or without DEX treatment (Supplementary Fig. 8c). The primary tumour sizes were slightly decreased in the DEX–GFP group (Supplementary Fig. 8c), similar to the results we found in miR-708-expressing tumours (Supplementary Fig. 1i). Individual organ metastasis was determined by the operator’s observation (Supplementary Fig. 9a), or by quantification of the hGAPDH levels (Fig. 6c). In agreement with the results we found in the miR-708-expressing tumour model (Fig. 1i), we observed reduced metastasis in various abdominal organs in the DEX–GFP group, including the liver, omentum, GI tract and diaphragm (Fig. 6c). Rap1B expression reversed DEX-mediated inhibition of metastasis in the liver, omentum and GI tract (Fig. 6c). The incidence of diaphragm metastasis was similar between PBS–GFP and Rap1B groups with or without DEX treatment (Supplementary Fig. 9a); however, the diaphragm-metastatic tumours were reduced in sizes in Rap1B groups with or without DEX treatment (Supplementary Fig. 9b, c), implying that the microenvironment of the diaphragm may not be suitable for Rap1B-mediated tumour growth. To further study gene regulation by DEX treatment *in vivo*, levels of miR-708 and its downstream molecules were examined from the orthotopically implanted primary ovarian tumours. Expressions of miR-708 were induced to over sevenfolds by DEX in both GFP and Rap1B groups, as compared with the GFP–PBS group (Fig. 6d). Western blot analysis from orthotopic primary ovarian tumours revealed that DEX treatment reduced the Rap1B protein level in the GFP–DEX group (Fig. 6e,f), correlated with our *in vitro* observation that expression of Rap1B was reduced in the presence of DEX (Fig. 3g). Overall, we demonstrate for the first time that GC-mediated signalling inhibits ovarian cancer abdominal metastasis, and expression of Rap1B significantly restored metastasis from this inhibition.

### Rap1B expression shows negative correlation with miR-708

**Figure 7:**
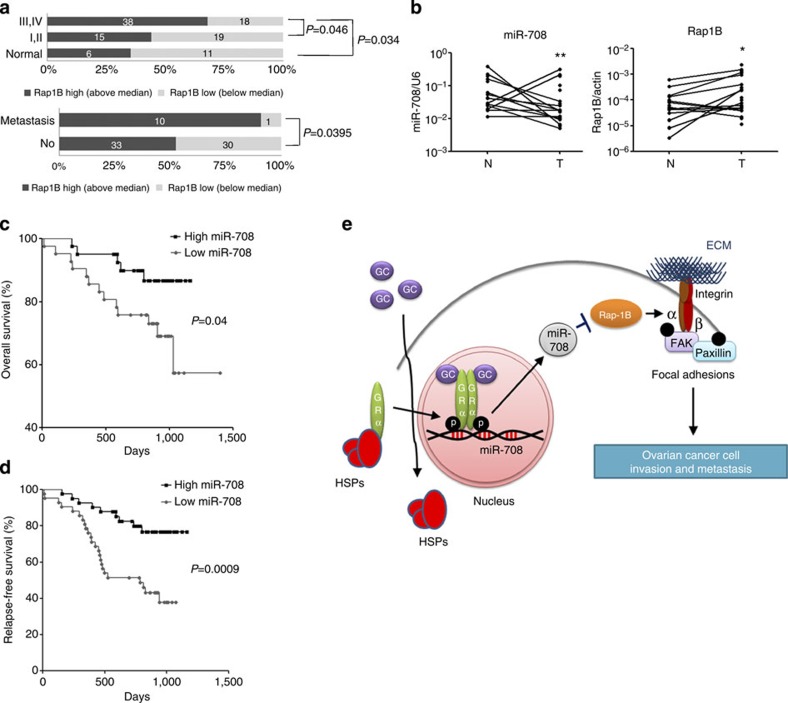
Rap1B shows negative correlation with miR-708, and patients with high miR-708 expression show better survival. (**a**) Rap1B mRNA expression in primary tumours using real-time PCR analysis from commercial cDNA arrays and TVGH-frozen samples. Samples were further subgrouped by tumor stages or whether metastasis occurred. The statistical significance was determined using *χ*^2^ test. (**b**) Real-time PCR analysis of N/T (normal/tumour) pairs, plus several unmatched N or T samples, from TVGH clinical frozen samples. MicroRNA-708 and Rap1B expression profiles were analysed. Student’s *t*-test (two-tailed) was used for statistical analysis (*P<0.05; **P<0.01). (**c**,**d**) Retrospective analysis of Kaplan–Meier plots for miRNA-708 expression in association with overall (**c**) or relapse-free (**d**) survival from 82 patients. Patients were split into high and low expression groups based on the median expression of the miR-708 (*n*=82; log-rank test). (**e**) Model of ovarian cancer metastasis suppressed via GC-mediated signalling pathways.

In order to evaluate the clinical relevance of Rap1B in ovarian cancer progression, we analysed its expression in commercial cDNA arrays and frozen tissues obtained from Taipei Veterans General Hospital (TVGH). Since Rap1B shared 95% amino-acid identity with Rap1A, the lack of differential antibodies for IHC forced us to limit our study at the RNA levels. The *Rap1B* level in primary tumours was significantly increased in patients with advanced stages of this disease (III/IV) or harbouring metastasis, as compared with normal ovary tissues or early-stage tumours (I/II) (Fig. 7a), and this pattern was opposite to that of miR-708 (Fig. 1b,c). We further analysed miRNA-708 and *Rap1B* expressions in 13 pairs of ovarian cancer carcinoma (T) with matched adjacent nontumour tissue (N), plus several unmatched N or T samples from TVGH (Supplementary Table 4). The miR-708 expression was significantly decreased in tumour samples, while expression of *Rap1B* was increased (Fig. 7b), suggesting an inverse correlation between *Rap1B* and miR-708 expression.

### MiRNA-708 expression is associated with patient survival

To further evaluate the clinical significance of miR-708 in ovarian cancer, 82 paraffin-embedded primary ovarian tumour specimens obtained from TVGH were analysed (Supplementary Table 5). As compared with low miR-708, patients with high miR-708 expression showed a significant better overall survival and relapse-free survival rates (Fig. 7c,d), and this difference is statistically significant in the advanced (stage III/IV), but not in the early (stage I/II), stage diseases (Supplementary Fig. 10), suggesting that miR-708 may have prognostic and therapeutic potentials for the future applications in the treatment of ovarian cancer.

## Discussion

In this study, we have unveiled a novel regulatory mechanism by steroid hormones, GCs, and its effect on ovarian cancer metastasis. Signalling mediated by GCs induced miR-708 expression, leading to the suppression of Rap1B, the impairment of integrin-mediated FA formation, and suppression of ovarian cancer metastasis (Fig. 7e). Our study implies the potential use of GCs or its downstream mediators to inhibit cancer metastasis.

Previous studies presented controversial results in the use of GCs in ovarian cancers with respect to cancer cell apoptosis, survival and invasion as alluded to in the Introduction. Here we found that GC treatments reduced ovarian cancer metastasis through miR-708, unveiling a new potential of GC application in ovarian cancer therapy. We also observed an interesting phenomenon that treatments with higher concentration of DEX (20 μg per mouse each time)^7^ showed neither inhibition of tumour growth and abdominal metastasis, nor induction of miR-708, suggesting the importance of the dosage of GCs given. Future studies are needed to elucidate the optimal timing, duration, dosage, as well as the choice of appropriate GCs to improve the treatment regimen.

In this study, we have identified the involvement of Rap1B in ovarian cancer metastasis. Given the high sequence identity (95%) between Rap1A and Rap1B, we surprisingly found that knockdown of Rap1A had no effects on ovarian cancer cell migration/invasion (Fig. 3f), suggesting the functional specificity of the two isoforms. Although the relative contribution of each isoform was not well discussed in earlier lines of work, several reports have revealed this nonredundant relationship between Rap1A and Rap1B. Isoform-specific knockout mice present different phenotypes^30^,^31^. Rap1B-null mice are smaller in size and decreased integrin-mediated cell adhesion was observed. Moreover, Rap1A regulates endothelial cell junction through E-Cadherin^32^, while Rap1B determines neuronal polarity through sequential activation of Cdc42 (ref. 33). Rap1A is predoominantly localized at the cell junctions, whereas Rap1B is more cytosolic and perinuclearly localized in endothelial cells^32^. Given the differential subcellular localizations, it is tempting to speculate that Rap1A and Rap1B may have distinct interacting complexes inside the cell.

To date, the role of Rap1 in the regulation of cell motility is still under debates. It has been shown that Rap1 activation by different Rap1 regulators, such as Rap1 guanine exchange factors (GEFs) or GTPase-activator proteins (GAPs), can either promote or inhibit cell motility and, moreover, to promote or inhibit metastasis in different cancer models^26^,^34^,^35^,^36^,^37^,^38^,^39^. Most studies so far mainly focused on controlling Rap1 activity by targeting distinct Rap1 GEFs or GAPs, such as Epac1 (refs 34, 36), DOCK4 (ref. 40), RapGEF2 (ref. 37) or Rap1GAP^39^, which may affect different pools of Rap1 at differential spatial locations and, consequently, lead to different outcomes. Here our data showed that suppression of Rap1B by miR-708 resulted in the decreased migration/invasion of ovarian cancer cells (Fig. 3). Downregulation of Rap1 by other miRNAs was also shown to inhibit cell invasion in breast cancer^27^ and oesophageal squamous cell carcinoma^41^. On the basis of those observations, we think that miRNA-mediated diminishment of Rap1 decreased its overall level, which in turn overrode spatial or temporal regulation of Rap1 signalling by different Rap1 regulators. Similarly, overexpression of Rap1 could also bypass the specific regulation of this molecule both spatially and temporally. Namely, while regulation of Rap1 by its GEFs or GAPs could have distinct effect of differential subcellularly localized Rap1, miRNAs-mediated regulation of the molecule will be global decrease. Furthermore, with respect to the distinct isoforms, Rap1A and Rap1B, our data revealed that only depletion of Rap1B, but not Rap1A, significantly inhibited migration/invasion of ovarian cancer cells (Fig. 3f), suggesting the dominant role of Rap1B in promoting migration/invasion. We propose that miRNA-708-mediated suppression of Rap1B plays an important role in inhibiting ovarian cancer cell migration/invasion and metastasis.

In summary, our study provides a new indication of GCs such as DEX for inhibition of ovarian cancer metastasis. It will be important to develop optimal regimen of GCs in terms of dosage and timing of treatments in combination with conventional therapies of ovarian cancer to obtain the maximal therapeutic benefits especially in the intervention of metastasis.

## Methods

### Cell culture and transfections

The human ovarian cancer cell lines SKOV-3 and TOV-112D were from ATCC (Manassas, VA). The A1847 and A2780 cells were obtained from Dr Stuart Aaronson (Mount Sinai School of Medicine). The selected invasive lines, SKOV-I6iv, TOV-I4, A1847-I4 and A2780-I4, have been described previously^15^. All cell lines were cultured in DMEM (Invitrogen) supplemented with 10% fetal bovine serum (FBS; Biological Industries, Israel) at 37 °C in 5% CO_2_. Cells were transfected using Lipofectamine 2000 (Invitrogen). Twenty nanomolars were used for each miRNA, anti-miRNA or siRNA transfection. SKOV-I6iv cell lines stably transfected with pSuper-GFP-Luc or pSuper-GFP-Luc-miR-708 were established by selection with G418 (Sigma-Aldrich). Three individual clones were mixed in equal proportion as stable clones. The SKOV-I6iv-pSuper-GFP-Luc stable cells were further infected with lentiviral particles encoding GFP or Rap1B genes. These two stable lines were selected from mass culture by puromycin (Sigma-Aldrich).

### Human samples

Frozen or paraffin-embedded ovarian tumour samples were obtained from the Department of Pathology, Taipei Veterans General Hospital, under the approved IRB protocol. Informed written consent was obtained from all patients who participated in the study. Tumour samples were collected during debulking surgery and patient’s identity of pathological specimens remained anonymous. Detailed information is provided in Supplementary Tables 4 and 5. Commercial tissue array slides were purchased from US Biomax (Rockville, MD) and SUPER BIO CHIPS (Seoul, South Korea). Commercial cDNA tissue array were purchased from OriGene Technologies Inc. (Rockville).

### Nucleotides and reagents

The human Rap1B was amplified through PCR from cDNA of SKOV-3 cells, and cloned into the pCMV-HA vector. For miR-708-expressing vector, 88 bps of miR-708 precursor was cloned into the pSuper plasmid with GFP-Luciferase fusion protein (pSuper-GFP-Luc). For Rap1B-expressing vector, full-length Rap1B was amplified and cloned into pLKO.EGFP.puro lentiviral construct (national RNAi core facility platform, Taiwan) by replacing GFP to Rap1B. The precursor miR-708 was from Applied Biosystems (Carlsbad, CA), and anti-miR-708 was from GeneDireX (Vegas, NV). The GRα-, ODZ4-, Rap1A- and Rap1B-specific siRNA were from MDBio Inc (QingDao, China). Detailed sequences are listed in Supplementary Table 1. DEX, prednisolone and cycloheximide were from Sigma-Aldrich, and MG132 was from R&D Systems.

### Quantitative real-time PCR for genes and miRNAs

RNA was extracted from cells using TRIzol (Invitrogen) following protocols supplied by the manufacturer. First-strand cDNA was generated by ReverTraAce (Toyobo, Japan) using oligo-dT or corresponding miRNA RT primers. Real-time RT–PCR was performed on a CFX96 real-time PCR detection system (Bio-Rad). The KAPA SYBR FAST Universal qPCR Kit (KAPA Biosystems, MA) was used for gene detection. The KAPA PROBE FAST universal qPCR Kit together with Universal Probe Library no. 21 (Roche Applied Science) were used for miRNA detection. The mRNA levels were normalized with actin, while miRNA levels were normalized with U6. All the primer sequences used in this study are provided in Supplementary Table 1. Experiments were repeated at least three times.

### 3′ UTR luciferase reporter and luciferase promoter assay

In 3′ UTR reporter assay, SKOV-I6iv cells were transfected with the pGL3-Rap1B 3′ UTR (wild type (WT) or mutant), Renilla, together with miR-708 or miR-708 plus anti-miR-708. In promoter assay, SKOV-3 cells transfected with pGL4-Promo-1 to 4, together with Renilla, were treated with 1 μM DEX or DMSO for 24 or 48 h. Sequences containing predicted miR-708-binding sites (116 bps) on Rap1B mRNA 3′ UTR region (WT or mutant form) were cloned into pGL3-Basic Vector (Promega). Sequences containing predicted promoter regions of *ODZ4* were amplified and cloned into pGL4.20 (Promega). Primer sequences used are provided in Supplementary Table 1. Luciferase activity assay was then performed and normalized with Renilla activity (Promega). Experiments were repeated three times.

### ChIP assay

ChIP material was prepared in accordance with the Magna ChIP (Millipore) manufacturer’s guidelines. Briefly, 1 × 10^7^ SKOV-3 cells were treated with 1 μM DEX or DMSO control for 3 h, and then ChIP assay was performed. IP was applied using 1 μg GR antibody (Cell Signaling) and equivalent amount of Rabbit IgG and H3 antibody (Millipore). PCR primers were designed on the promoter-3 region of *ODZ4*, and primer sequences used are provided in Supplementary Table 1. The PCR products were visualized using agarose gel electrophoresis or quantitative PCR. Experiments were repeated two times.

### Western blot analysis

Cells were lysed in 10 mM Tris buffer, pH 7.4, containing 0.15 M NaCl, 1% Triton X-100, 1 mM EDTA and protease inhibitor mixture (Roche). The cell lysates were resolved on an 8–12% SDS–polyacrylamide gel, transferred on polyvinylidene difluoride membrane, and probed with antibodies. Antibodies against Rap1A/B, Rap1B, p-Paxillin (Tyr118), p-FAK (Tyr861), p-GR (Ser211), GR and HA-tag (C29F4) were from Cell Signaling Technology. Antibodies against FAK and Paxillin were from Millipore. Anti-actin and anti-GAPDH were from Santa Cruz Biotechnology. Protein expression levels were normalized to β-actin or GAPDH, and are presented as fold changes relative to the control. All experiments were repeated three times. Full scans of all western blots are included in Supplementary Fig. S11.

### Transwell cell migration and cell invasion assay

Cell migration and invasion boyden chamber assays were performed as previously described^42^. Cell migration was assayed in 8.0-mm Falcon Cell Culture Inserts (Corning), and, for the cell invasion assay, the BD biocoat matrigel invasion chamber was applied (Corning). Briefly, 5 × 10^4^ cells were suspended in DMEM (300 μl) and placed in the upper transwell of 0.3 cm^2^ in area. The bottom well was filled with 500 μl DMEM with 10% FBS. After incubation of 6–16 h (migration) or 8–24 h (invasion), cells on the upper side of the inserts were removed by cotton swabs, and cells on the underside were fixed and stained with crystal violet. Photos of three regions were taken from duplicated inserts, and the numbers of cells were counted using Image J (NIH, USA). All experiments were repeated three times.

### Wound-healing assay

Wound-healing assays were carried out by using migration culture dish inserts from Ibidi (Martinsried, Germany) or by using pipette tip to make a straight scratch. Cells were seeded in the chambers of the culture dish insert or 24-well plates and transfected with appropriate vectors. Forty-eight hours after transfection, cells were at full confluency, the insert was removed or a straight scratch was made by pipette tip, and the fresh culture medium was added to start the migration process. Pictures were acquired after 0–24 h using microscope with × 4 objective (IX51, Olympus). Image analysis was performed using Image J (NIH). For migration area quantification, the cell-free area of each picture from 0 h to the end point was measured manually, and the migrating area was defined as the cell-free area at 0 h minus the cell-free area at the end point. All experiments were repeated three times.

### Adhesion assay

SKOV-I6iv cells (2 × 10^4^) transfected with the GFP plasmid were plated on 24 wells pre-coated with FN (10 μg ml^−1^; Millipore) or collagen I (10 μg ml^−1^; BD) at 37 °C for 1 h. The cells were rinsed with PBS for five times to remove nonadherent cells, and images of the cells before and after washing were taken using fluorescent microscope with × 4 objective (IX51, Olympus). Ten fields were randomly selected, and the numbers of attached GFP-positive cells were counted. Data represent the average percentage of attached cells±s.d. from three independent experiments.

### Cell proliferation assay

Cell proliferation assay was measured with the CellTiter 96 Aqueous One Solution cell proliferation assay (Promega). Assay was performed according to the methods described in the manufacturer’s manual. Briefly, cells (1 × 10^3^ cells) were seeded in 96-well plates and incubated for various times, at defined time points, 20 μl of the CellTiter 96 AQueous One Solution Reagent was added and incubated for 2 h at 37 °C. The quantity of formazan product, which is directly proportional to the number of living cells in the culture, was measured by absorbance at 490 nm with 96-well plate reader. Data are the means±s.d. from three independent experiments.

### Fluorescence *in situ* hybridization and immunofluorescence

Biotin-labelled locked nucleic acid-modified RNA probes (GeneDireX), directed against the full-length mature miR-708 sequence, were used for miRNA detection. Streptavidin-horseradish peroxidase IgG and tyramide signal amplification reactions (PerkinElmer) were used to detect the hybridized probes. Immunofluorescence was carried out using antibodies against p-FAK, p-Paxillin, FAK, paxillin or HA-tag (Cell Signaling), or Rhodamine Phalloidin (Invitrogen) in 1:100 dilution, and Alexa Fluor 488, 594 or 647 coupled antibodies (Invitrogen) were used as the secondary antibodies in 1:500 dilution. The slides were mounted and images were captured using fluorescence microscopy (IX51, Olympus; or DM2500, Leica) or confocal microscopy (TCS-SP5, Leica). The intensities of fluorescence signals in the tumour regions were quantified using Image J.

### Assay for FA and cell spreading

SKOV-I6iv cells were trypsinized, plated on fibronection (10 μg ml^−1^; Millipore)-coated coverslips and incubated at 37 °C for 90 min in DMEM. According to the methods described by in ref. 43, FAs were detected with anti-paxillin antibody, and actin was visualized using Rhodamine Phalloidin (Invitrogen). Images were acquired using confocal microscopy (TCS SP5, Leica) with × 63 objective lenses. Image J was used to quantify cell spreading and FA staining. At least 30 cells per condition were analysed. To avoid bias, all of the cells imaged from a single field of view were analysed. Images were taken from two or three independent experiments and combined together. Cells were selected using the threshold function, to measure either the whole area of the cell, or the areas stained for FAs. FAs were analysed by measuring the total area per cell that stained for paxillin, and was standardized to the mean area of FA staining measured under control conditions. Total areas of each cell were measured from phalloidin staining and the spreading index was determined by standardizing to the mean size of cells under control conditions. Data are presented as the means±s.e.m.

### Mouse tail–vein injection and orthotopic metastasis model

In tail–vein injection, SKOV-I6iv cells (1 × 10^6^) with different gene modifications were harvested and re-suspended in 100 μl PBS. The cells were injected intravenously through the tail vein into 6- to 8-week-old CB17/lcr-Prkdc scid/Crl mice (BioLasco, Taiwan). All the mice were killed 3 weeks after injection. The lungs of mice were harvested, fixed with formalin, sectioned and subjected to haematoxylin and eosine (H&E) staining. Ten pictures were captured from each H&E-stained slide, and total areas of metastatic nodules per field were counted using Image J. In the orthotopic implantation, SKOV-I6iv cells (1 × 10^6^) with different gene modifications were harvested and re-suspended in 20 μl PBS containing 50% Matrigel (BD Biosciences). Cells were injected intrabursally into the ovary of 6- to 8-week-old CB17/lcr-Prkdc scid/Crl mice. In the experiments with DEX treatment, 5 mg ml^−1^ DEX was dissolved in ethanol and further diluted in PBS for injection of 50 μg kg^−1^ per time, and then intraperitoneally injected into mice three times a week starting from day 5 after cell implantation. Tumour growths and abdominal metastases were monitored by live animal BLI (Caliper IVIS system, PerkinElmer). Abdominal metastases were quantified by total BLI signals of each mouse minus primary tumour BLI signals (Supplementary Fig. 1f). Mice were killed after 30 days of implantation. Abdominal organs were collected and homogenized in Trizol (Invitrogen) by TissueLyser II (QIAGEN). Micrometastases in different organs were quantified using real-time RT–PCR by detecting human-specific GAPDH levels and normalized to universal β-actin levels. All procedures were carried out under approved Institutional Animal Care and Use Committee protocols.

## Author contributions

K.-T.L., Y.-M.Y., S.Y.Y., J.-W.C., S.-P.S. and Y.-S.W. carried out the experimental work. C.-M.C. and K.-C.C. provided and assisted in the clinical sample analysis. K.-T.L. designed experiments and wrote the manuscript. L.-H.W. obtained funding for this project, directed and supervised the research, as well as revised and approved the manuscript. K.-C.C. and L.-H.W. are co-corresponding authors.

## Additional information

**How to cite this article**: Lin, K.-T. *et al.* Glucocorticoids mediate induction of microRNA-708 to suppress ovarian cancer metastasis through targeting Rap1B. *Nat. Commun.* 6:5917 doi: 10.1038/ncomms6917 (2015).

## Supplementary Material

Supplementary Information Supplementary Figures 1-11 and Supplementary Tables 1-5.

## References

[b1] Jemal A. *et al.* Global cancer statistics. CA Cancer J. Clin. 61, 69–90 (2011). 21296855 10.3322/caac.20107

[b2] Cannistra S. A. Cancer of the ovary. N. Engl. J. Med. 351, 2519–2529 (2004). 15590954 10.1056/NEJMra041842

[b3] Hennessy B. T., Coleman R. L. & Markman M. Ovarian cancer. Lancet 374, 1371–1382 (2009). 19793610 10.1016/S0140-6736(09)61338-6

[b4] Gennari A., Salvadori B., Tognoni A. & Conte P. F. Rapid intravenous premedication with dexamethasone prevents hypersensitivity reactions to paclitaxel. Ann. Oncol, 7, 978–979 (1996). 9006754 10.1093/oxfordjournals.annonc.a010806

[b5] Runnebaum I. B. & Bruning A. Glucocorticoids inhibit cell death in ovarian cancer and up-regulate caspase inhibitor cIAP2. Clin. Cancer Res. 11, 6325–6332 (2005). 16144937 10.1158/1078-0432.CCR-05-0182

[b6] Zhang C. *et al.* Glucocorticoid-mediated inhibition of chemotherapy in ovarian carcinomas. Int. J. Oncol. 28, 551–558 (2006). 16391812

[b7] Sui M., Chen F., Chen Z. & Fan W. Glucocorticoids interfere with therapeutic efficacy of paclitaxel against human breast and ovarian xenograft tumors. Int. J. Cancer 119, 712–717 (2006). 16496381 10.1002/ijc.21743

[b8] Munstedt K., Borces D., Bohlmann M. K., Zygmunt M. & von Georgi R. Glucocorticoid administration in antiemetic therapy: is it safe? Cancer 101, 1696–1702 (2004). 15468188 10.1002/cncr.20534

[b9] Rubenstein N. M., Guan Y., Woo P. L. & Firestone G. L. Glucocorticoid down-regulation of RhoA is required for the steroid-induced organization of the junctional complex and tight junction formation in rat mammary epithelial tumor cells. J. Biol. Chem. 278, 10353–10360 (2003). 12525486 10.1074/jbc.M213121200

[b10] Zheng Y., Izumi K., Li Y., Ishiguro H. & Miyamoto H. Contrary regulation of bladder cancer cell proliferation and invasion by dexamethasone-mediated glucocorticoid receptor signals. Mol. Cancer Ther. 11, 2621–2632 (2012). 23033490 10.1158/1535-7163.MCT-12-0621

[b11] Law M. E. *et al.* Glucocorticoids and histone deacetylase inhibitors cooperate to block the invasiveness of basal-like breast cancer cells through novel mechanisms. Oncogene 32, 1316–1329 (2013). 22543582 10.1038/onc.2012.138 PMC3773700

[b12] Saini S. *et al.* MicroRNA-708 induces apoptosis and suppresses tumorigenicity in renal cancer cells. Cancer Res. 71, 6208–6219 (2011). 21852381 10.1158/0008-5472.CAN-11-0073 PMC3940359

[b13] Saini S. *et al.* miRNA-708 control of CD44(+) prostate cancer-initiating cells. Cancer Res. 72, 3618–3630 (2012). 22552290 10.1158/0008-5472.CAN-12-0540

[b14] Ryu S. *et al.* Suppression of miRNA-708 by polycomb group promotes metastases by calcium-induced cell migration. Cancer Cell 23, 63–76 (2013). 23328481 10.1016/j.ccr.2012.11.019

[b15] Yeh Y. M., Chuang C. M., Chao K. C. & Wang L. H. MicroRNA-138 suppresses ovarian cancer cell invasion and metastasis by targeting SOX4 and HIF-1alpha. Int. J. Cancer (2013). 10.1002/ijc.28086 23389731

[b16] Hwang S. J. *et al.* MicroRNA-146a suppresses metastatic activity in brain metastasis. Mol. Cell 34, 329–334 (2012). 10.1007/s10059-012-0171-6 PMC3887840 22949171

[b17] Zhang H. F., Xu L. Y. & Li E. M. A family of pleiotropically acting microRNAs in cancer progression, miR-200: potential cancer therapeutic targets. Curr. Pharm. Design 20, 1896–1903 (2013). 10.2174/13816128113199990519 23888967

[b18] Wu D. W., Cheng Y. W., Wang J., Chen C. Y. & Lee H. Paxillin predicts survival and relapse in non-small cell lung cancer by microRNA-218 targeting. Cancer Res. 70, 10392–10401 (2010). 21159652 10.1158/0008-5472.CAN-10-2341

[b19] Han B. W. *et al.* A set of miRNAs that involve in the pathways of drug resistance and leukemic stem-cell differentiation is associated with the risk of relapse and glucocorticoid response in childhood ALL. Hum. Mol. Genet. 20, 4903–4915 (2011). 21926415 10.1093/hmg/ddr428 PMC3221537

[b20] Behrman S., Acosta-Alvear D. & Walter P. A CHOP-regulated microRNA controls rhodopsin expression. J. Cell Biol. 192, 919–927 (2011). 21402790 10.1083/jcb.201010055 PMC3063143

[b21] Kinbara K., Goldfinger L. E., Hansen M., Chou F. L. & Ginsberg M. H. Ras GTPases: integrins’ friends or foes? *Nature reviews*. Mol. Cell Biol. 4, 767–776 (2003). 10.1038/nrm1229 14570053

[b22] Boettner B. & Van Aelst L. Control of cell adhesion dynamics by Rap1 signaling. Curr. Opin. Cell Biol. 21, 684–693 (2009). 19615876 10.1016/j.ceb.2009.06.004 PMC2841981

[b23] Bos J. L. Linking Rap to cell adhesion. Curr. Opin. Cell Biol. 17, 123–128 (2005). 15780587 10.1016/j.ceb.2005.02.009

[b24] Robin T. P. *et al.* EWS/FLI1 regulates EYA3 in Ewing sarcoma via modulation of miRNA-708, resulting in increased cell survival and chemoresistance. Mol. Cancer Res. 10, 1098–1108 (2012). 22723308 10.1158/1541-7786.MCR-12-0086 PMC3432289

[b25] Bokoch G. M. Biology of the Rap proteins, members of the ras superfamily of GTP-binding proteins. Biochem. J. 289, 17–24 (1993). 8424755 10.1042/bj2890017 PMC1132124

[b26] Huang M. *et al.* EGFR-dependent pancreatic carcinoma cell metastasis through Rap1 activation. Oncogene 31, 2783–2793 (2012). 21963850 10.1038/onc.2011.450 PMC3711644

[b27] Bischoff A. *et al.* miR-149 functions as a tumor suppressor by controlling breast epithelial cell migration and invasion. Cancer Res. 74, 5256–5265 (2014). 25035394 10.1158/0008-5472.CAN-13-3319

[b28] Benlimame N. *et al.* FAK signaling is critical for ErbB-2/ErbB-3 receptor cooperation for oncogenic transformation and invasion. J. Cell Biol. 171, 505–516 (2005). 16275754 10.1083/jcb.200504124 PMC2171271

[b29] Nakamura K., Yano H., Schaefer E. & Sabe H. Different modes and qualities of tyrosine phosphorylation of Fak and Pyk2 during epithelial-mesenchymal transdifferentiation and cell migration: analysis of specific phosphorylation events using site-directed antibodies. Oncogene 20, 2626–2635 (2001). 11420674 10.1038/sj.onc.1204359

[b30] Duchniewicz M. *et al.* Rap1A-deficient T and B cells show impaired integrin-mediated cell adhesion. Mol. Cell Biol. 26, 643–653 (2006). 16382154 10.1128/MCB.26.2.643-653.2006 PMC1346907

[b31] Chrzanowska-Wodnicka M., Kraus A. E., Gale D., White G. C. 2nd & Vansluys J. Defective angiogenesis, endothelial migration, proliferation, and MAPK signaling in Rap1b-deficient mice. Blood 111, 2647–2656 (2008). 17993608 10.1182/blood-2007-08-109710 PMC2254536

[b32] Wittchen E. S., Aghajanian A. & Burridge K. Isoform-specific differences between Rap1A and Rap1B GTPases in the formation of endothelial cell junctions. Small GTPases 2, 65–76 (2011). 21776404 10.4161/sgtp.2.2.15735 PMC3136906

[b33] Schwamborn J. C. & Puschel A. W. The sequential activity of the GTPases Rap1B and Cdc42 determines neuronal polarity. Nat. Neurosci. 7, 923–929 (2004). 15286792 10.1038/nn1295

[b34] Lyle K. S., Raaijmakers J. H., Bruinsma W. & Bos J. L. & de Rooij, J. cAMP-induced Epac-Rap activation inhibits epithelial cell migration by modulating focal adhesion and leading edge dynamics. Cell. Signal. 20, 1104–1116 (2008). 18346875 10.1016/j.cellsig.2008.01.018

[b35] Freeman S. A. *et al.* Preventing the activation or cycling of the Rap1 GTPase alters adhesion and cytoskeletal dynamics and blocks metastatic melanoma cell extravasation into the lungs. Cancer Res. 70, 4590–4601 (2010). 20484042 10.1158/0008-5472.CAN-09-3414

[b36] Gao L. *et al.* Ras-associated protein-1 regulates extracellular signal-regulated kinase activation and migration in melanoma cells: two processes important to melanoma tumorigenesis and metastasis. Cancer Res. 66, 7880–7888 (2006). 16912161 10.1158/0008-5472.CAN-06-0254

[b37] Magliozzi R. *et al.* Control of epithelial cell migration and invasion by the IKKbeta- and CK1alpha-mediated degradation of RAPGEF2. Dev. Cell 27, 574–585 (2013). 24290981 10.1016/j.devcel.2013.10.023

[b38] Hernandez-Varas P. *et al.* Rap1-GTP-interacting adaptor molecule (RIAM) protein controls invasion and growth of melanoma cells. J. Biol. Chem. 286, 18492–18504 (2011). 21454517 10.1074/jbc.M110.189811 PMC3099666

[b39] Kim W. J., Gersey Z. & Daaka Y. Rap1GAP regulates renal cell carcinoma invasion. Cancer Lett. 320, 65–71 (2012). 22266190 10.1016/j.canlet.2012.01.022 PMC3319804

[b40] Yajnik V. *et al.* DOCK4, a GTPase activator, is disrupted during tumorigenesis. Cell 112, 673–684 (2003). 12628187 10.1016/s0092-8674(03)00155-7

[b41] Zhang M. *et al.* miR-518b is down-regulated, and involved in cell proliferation and invasion by targeting Rap1b in esophageal squamous cell carcinoma. FEBS Lett. 586, 3508–3521 (2012). 22958893 10.1016/j.febslet.2012.08.007

[b42] Sachdev P., Zeng L. & Wang L. H. Distinct role of phosphatidylinositol 3-kinase and Rho family GTPases in Vav3-induced cell transformation, cell motility, and morphological changes. J. Biol. Chem. 277, 17638–17648 (2002). 11884391 10.1074/jbc.M111575200

[b43] Ross S. H. *et al.* Ezrin is required for efficient Rap1-induced cell spreading. J. Cell Sci. 124, 1808–1818 (2011). 21540295 10.1242/jcs.079830

