# Supplementary material for: Glucocorticoids mediate induction of microRNA-708 to suppress ovarian cancer metastasis through targeting Rap1B
Source: Nat Commun. 2015 Jan 8;6:5917. doi: 10.1038/ncomms6917 (PMC4354140; doi:10.1038/ncomms6917)
Supplement: Supplementary Information — Supplementary Figures 1-11 and Supplementary Tables 1-5. [file ncomms6917-s1.pdf]

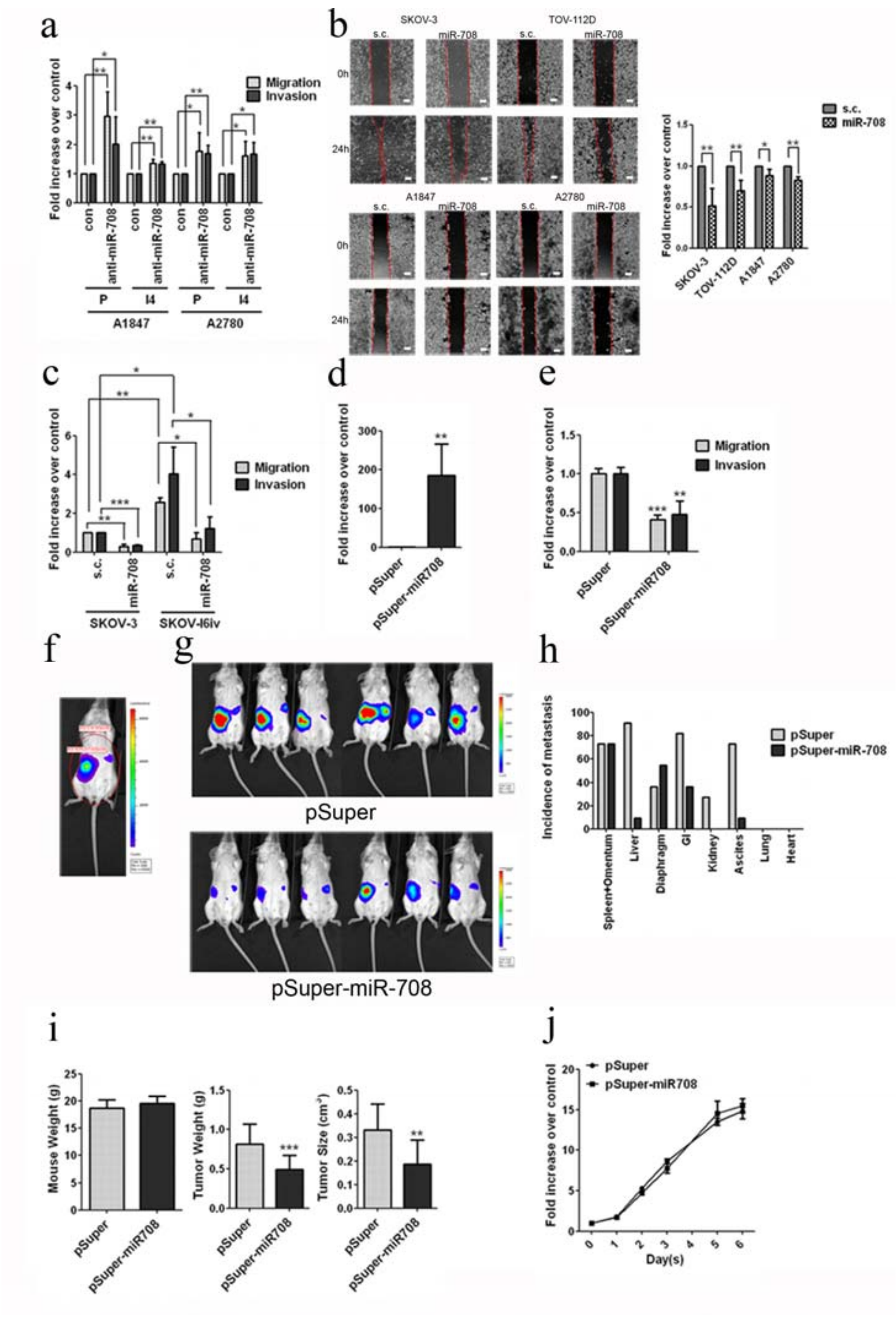

Supplementary Figure 1. Overexpression of miR-708 inhibits ovarian cancer cells migration/invasion and metastasis. (a) A1847, A1847-I4, A2780, and A2780-I4 cells were transfected with anti-miR-708 or control. Migration (16h) and invasion (24h) assays were performed after 24 h. DMEM/10% FBS served as the chemoattractant. Numbers represent normalized means ± SD (n = 3). (b) Left: Wound-healing assay of SKOV-3, TOV-112D, A1847, and A2780 cells transfected with precursor miR-708 or s.c. (scramble control). Images of the migration area were captured at 0 h and 24 h after removal of the culture insert. The red lines indicate the boundaries of the

wound. Scale bar: 200μM. Right: Quantitative results showing the relative migrating area compared to the control condition. Number represent normalized means ± SD (n = 3). (c) SKOV-3, and SKOV-I6iv cells were transfected with precursor miR-708 or s.c.. Migration (8h) and invasion (16h) assays were performed after 24 h. DMEM/10% FBS served as the chemoattractant. Numbers represent normalized means ± SD (n = 3). (d) Real time RT-PCR analysis of miRNA-708 levels in SKOV-I6iv cells stably expressing pSuper-Luc-GFP-miR-708 or vector control. Data are presented as normalized means ± SD (n = 3). (e) SKOV-I6iv cells stably expressing pSuper-Luc-GFP-miR-708 or vector control were assayed for migration/invasion. Cells were incubated 8 h for migration, and 16 h for invasion. DMEM/10% FBS served as a chemoattractant. Numbers represent normalized means ± SD (n = 3). (f) The schematic showing quantification methodology for abdominal metastasis. The whole abdomen of one mouse was circled and measured as total BLI signals. The smaller circle indicates signals from the primary tumor. The abdominal metastasis is considered as total BLI signals minus BLI signals from the primary tumor. (g) Bioluminescent images of mice bearing SKOV-I6iv tumors transfected with either pSuper-Luc-GFP-miR-708 or vector controls are shown at day 28 after implantation. (h) Percentage of tumor incidence in various organs following 30 days of orthotopic implantation of SKOV-I6iv cells with the indicated stable gene expression. The tumor incidence was determined by macro metastasis observed from operator's eyes. (i) SKOV-I6iv cells with pSuper-Luc-GFP-miR-708 expression or vector control were orthotopically implanted into mouse ovary. The mouse weight (g), primary tumor weight (g), and tumor size (cm^3) were compared. Data are presented as normalized means ± SD (n = 10 per group; data were combined from two separate experiments). (j) Cell proliferation assay was performed in SKOV-I6iv cells stably expressing pSuper-Luc-GFP-miR-708 or vector control by MTS reagent. $1x10^3$ cells were cultured and assayed for proliferation rates in 96 wells from 0 to 6 days. Data are the means ± SD (n=3). *$p < 0.05$; **$p < 0.01$; ***$p<0.001$.

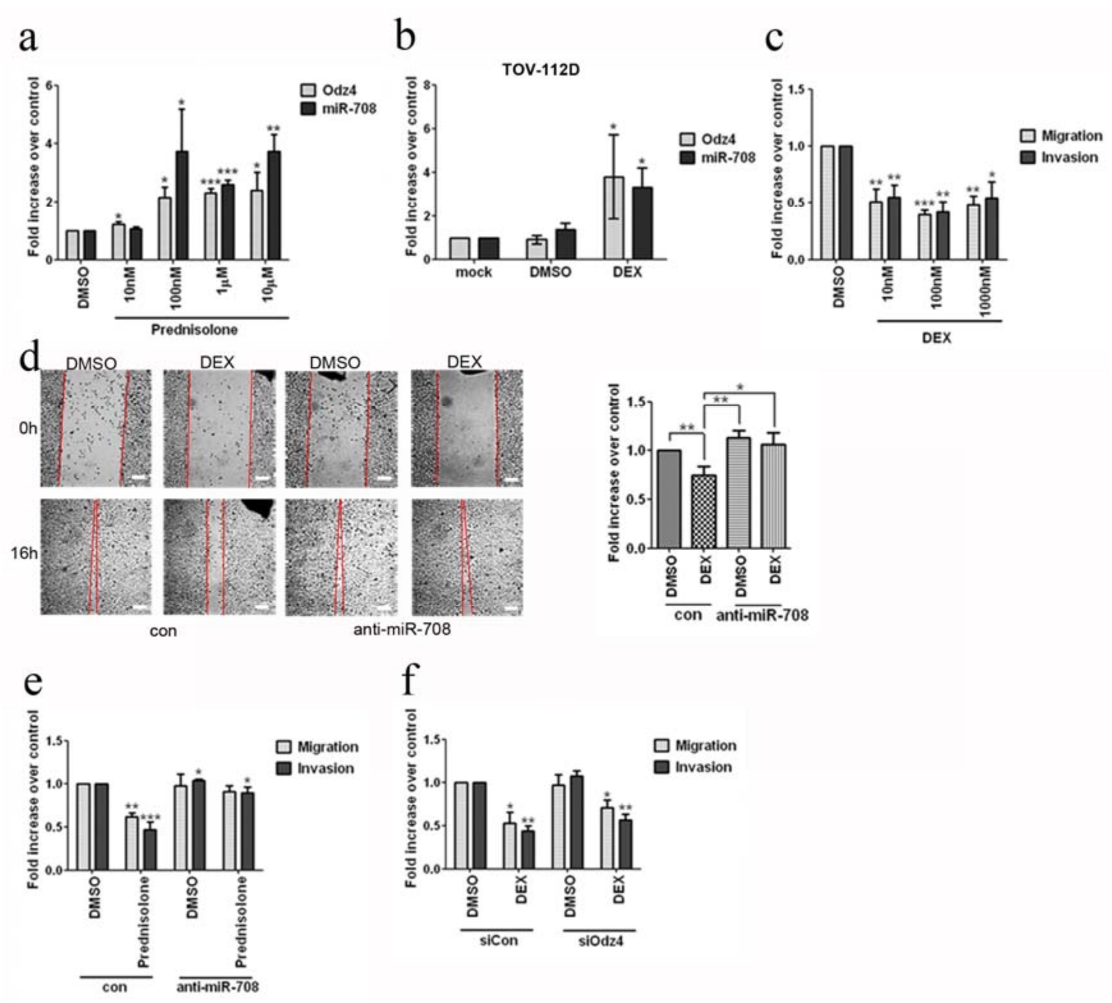

Supplementary Figure 2. Glucocorticoid-mediated signaling regulates miR-708 and *ODZ4* transcription in ovarian cancer cells. (a) Expression of *ODZ4* and miR-708 in SKOV-3 cells 72h after treatments with increasing amount of Prednisolone (10nM-10μM). Numbers represent normalized means ± SD (n = 3). (b) Expression of *ODZ4* and miR-708 in TOV-112D cells 72h after treatments with 1μM DEX. Data were normalized to mock levels. Numbers represent normalized means ± SD (n = 3). (c) SKOV-3 cells were pre-treated with 10nM, 100nM, 1μM DEX, or DMSO control overnight, and then incubated for migration (8h) and invasion (16h) assay. DMEM/10% FBS, together with various concentration of DEX or DMSO control, served as a chemoattractant. Numbers represent normalized means ± SD (n = 3). (d) Left: Wound-healing assay of SKOV-3 transfected with anti-miR-708 or control. The cells were pre-treated with 1μM DEX, or DMSO control overnight, and then incubated for migration assay in DMEM/10% FBS, together with DEX or DMSO control. Images of the migration area were captured at 0 h and 16 h after using the pipette tip to make a straight scratch. The red lines indicate the boundaries of the wound. Scale bar: 200μM. Right: Quantitative results showing the relative

migrating area compared to the control condition. Number represent normalized means ± SD (n = 3). (e) SKOV-3 cells transfected with anti-miR-708, or control, were treated with 1μM Prednisolone overnight, and then incubated for migration (8h) and invasion (16h) assay. DMEM/10% FBS, together with 1μM Prednisolone or DMSO control, served as a chemoattractant. Numbers represent normalized means ± SD (n = 3). (f) SKOV-3 cells transfected with ODZ4 or control siRNA were treated with 1μM DEX overnight, and then incubated for migration (8h) and invasion (16h) assay. DMEM/10% FBS, together with 1μM DEX or DMSO control, served as a chemoattractant. Numbers represent normalized means ± SD (n = 3).*$p < 0.05$; **$p < 0.01$; ***$p < 0.001$.

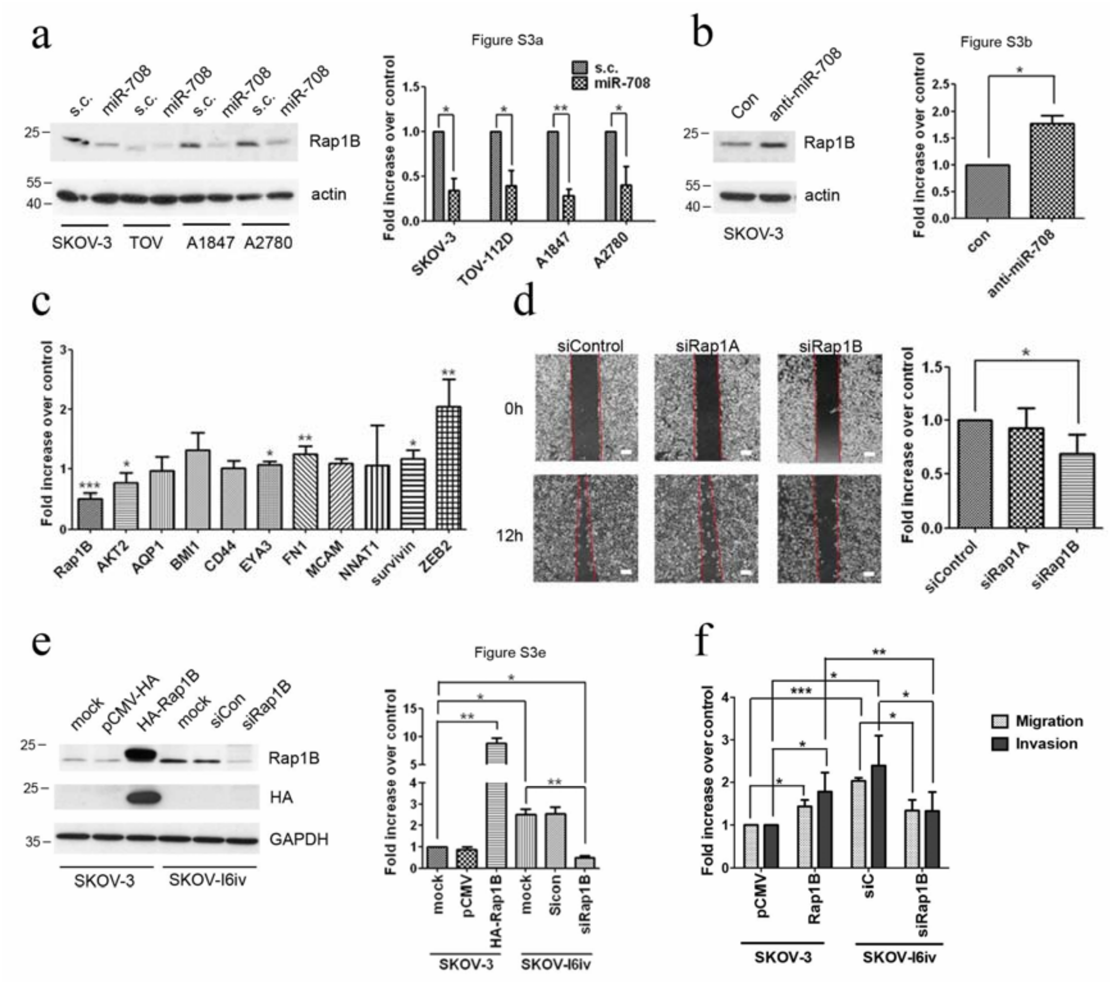

Supplementary Figure 3. Rap1B is the major target of miR-708 in ovarian cancer cells. (a) Left: Western blot analysis of Rap1B protein expression in SKOV-3, TOV-112D, A1847, and A2780 cells transfected with the miR-708 or scramble control (s.c.). Right: quantitative analysis of protein levels, and normalized with actin levels. Histograms represent normalized means ± SEM (n=3). (b) Left: Western blot analysis of Rap1B protein expressions in SKOV-3 cells transfected with the anti-miR-708 or control. Right: quantitative analysis of protein levels, and normalized with actin levels. Histograms represent normalized means ± SEM (n=3). (c) Real-time RT-PCR analysis showing the expression of potential miR-708 targeting genes in SKOV-I6iv cells after 48 h transfection with miR-708. Data were normalized to levels of s.c. group. Numbers represent normalized means ± SD (n = 3). (d) Left: Wound-healing assay of SKOV-I6iv cells transfected with Rap1A, Rap1B, and control siRNA. Images of the migration area were captured at 0 h and 12 h after removal of the culture insert. The red lines indicate the boundaries of the wound. Scale bar: 200μM. Right: Quantitative results showing the relative migrating area

compared to the control condition. Number represent normalized means ± SD (n = 3). (e) Left: Western blot analysis of Rap1B protein expression in SKOV-3 cells transfected with HA-tagged Rap1B or vector control, and SKOV-I6iv cells transfected with Rap1B and control siRNA. Right: quantitative analysis of Rap1B protein levels, and normalized with GAPDH levels. Histograms represent normalized means ± SEM (n=3). (f) SKOV-3 cells transfected with HA-tagged Rap1B or vector control and SKOV-I6iv cells transfected with Rap1B and control siRNA were incubated for migration (16h) and invasion (16h) assay. DMEM/10% FBS served as a chemoattractant. Numbers represent normalized means ± SD (n = 3).

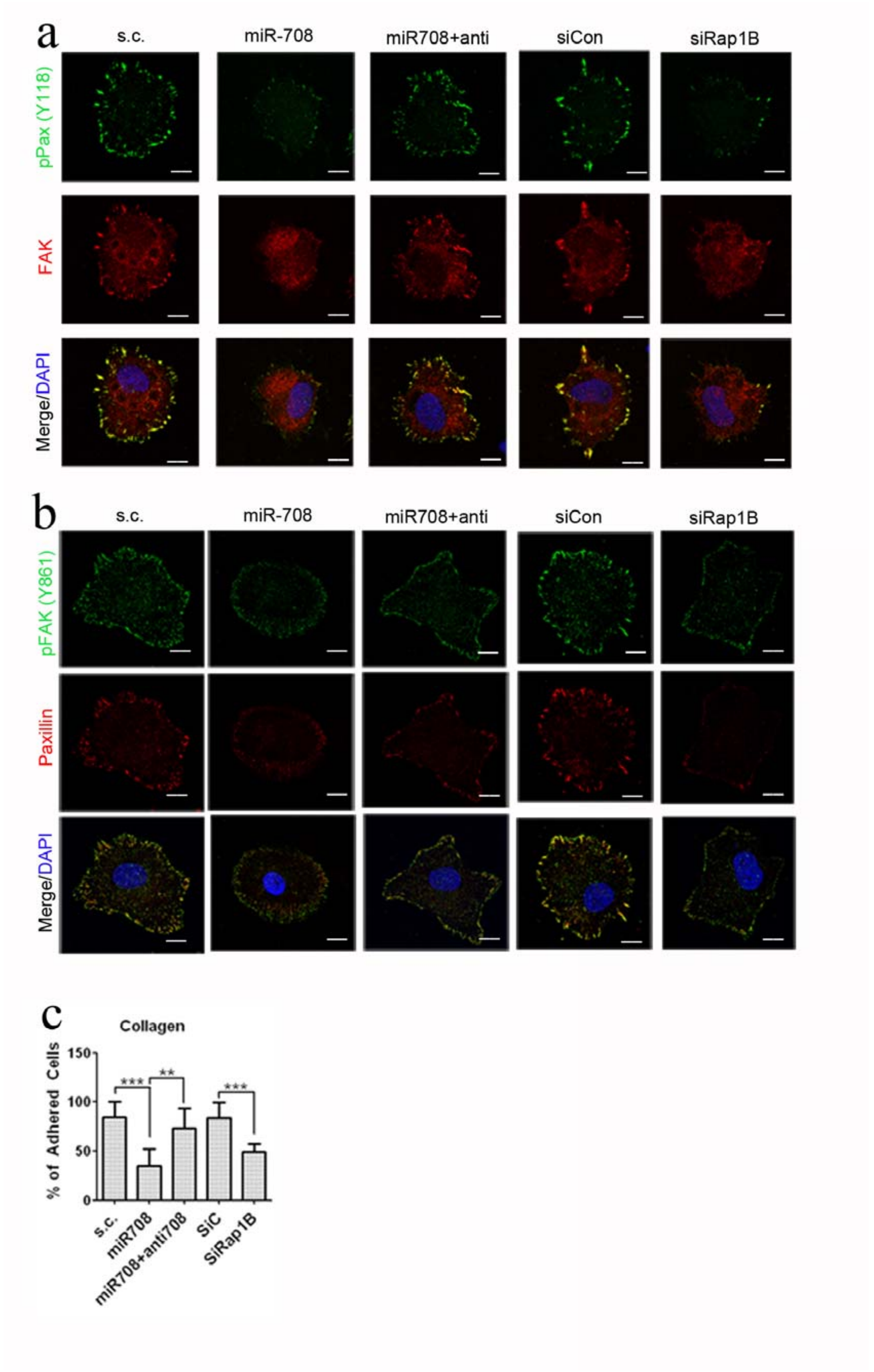

Supplementary Figure 4. MicroRNA-708 mediates integrin-regulated FA formation by targeting Rap1B. (a, b) Confocal microscopy of p-paxillin (Y118;

Green), total FAK (Red), and DAPI (Blue) staining (a), or p-FAK (Y861; Green), total paxillin (Red), and DAPI (Blue) staining (b), after adhering of cells to FN. SKOV-I6iv cells with different transfections were trypsinized, and then equally plated on FN-coated coverslips for 90 mins. Scale Bars: 10μm. (c) Adhesion assays of SKOV-I6iv cells transfected with GFP plus the indicated transfections. Cells were incubated on Collagen I-coated plates for 1h, and then washed with PBS 5 times. Percentage of adhered cells was counted, and numbers represent means ± SD (n=3). $^{**}p<0.01$; $^{***}p < 0.001$.

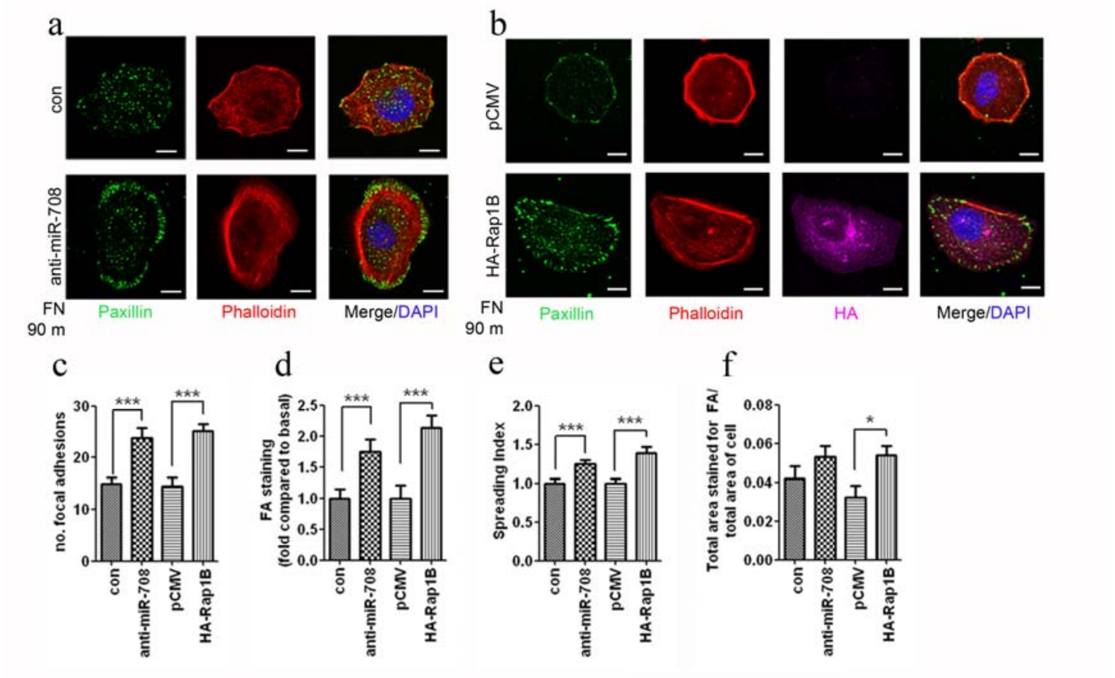

Supplementary Figure 5. Knockdown of miR-708 or expression of Rap1B in SKOV-3 parental cells stimulates cell spreading and FA formation (a, b) Confocal microscopy of Paxillin (Green), β-actin (Red), HA (Purple), and DAPI (Blue) nuclear staining, after adhering of cells to FN. SKOV-3 cells with anti-miR-708 or control (a), or pCMV-HA-Rap1B or vector control (b) were equally plated onto FN-coated coverslips for 90 mins. The FAs were detected. Scale Bars: 10μm. The images were analyzed to determine the number of FAs per cell (c), the area of the cell staining for FAs by paxillin staining (d), and the cell spreading area by β-actin staining (e). To determine the proportion of each cell consisting of FAs, the area of FAs was divided by the total spreading area of the cell (f). Data in (c-f) represent means ± SEM (n = 30). $*p < 0.05$; $***p < 0.001$.

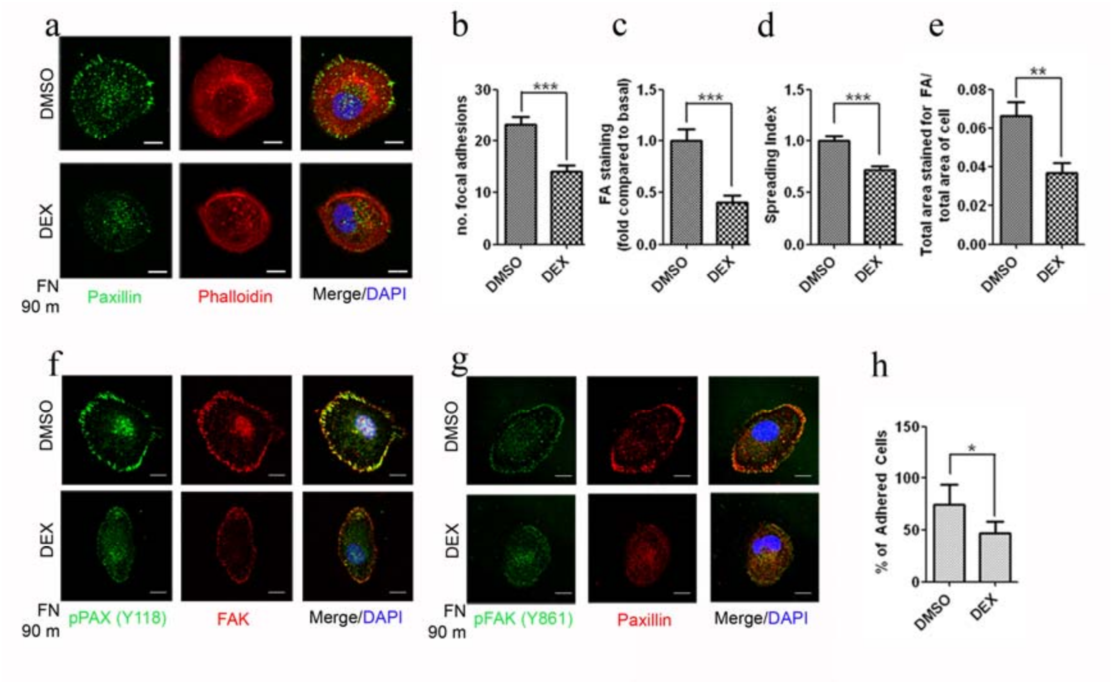

Supplementary Figure 6. Glucocorticoid-mediated signaling disrupts FA formation, cell spreading, and cell adhesion ability. (a) Confocal microscopy of Paxillin (Green), β-actin (Red), and DAPI (Blue) nuclear staining, after adhering of cells to FN. SKOV-I6iv cells were pre-treated with 1μM DEX or DMSO control for 24 h, and then equally plated onto FN-coated coverslips for 90 mins in the presence of 1μM DEX or DMSO. The FAs were detected. Scale Bars: 10μm. The images were analyzed to determine the number of FAs per cell (b), the area of the cell staining for FAs by paxillin staining (c), and the cell spreading area by β-actin staining (d). To determine the proportion of each cell consisting of FAs, the area of FAs was divided by the total spreading area of the cell (e). Data in (b-e) represent means ± SEM (n = 30). (f, g) Confocal microscopy of p-paxillin (Y118; Green), total FAK (Red), and DAPI (Blue) staining (f), or p-FAK (Y861; Green), total paxillin (Red), and DAPI (Blue) staining (g), after adhering of cells to FN. SKOV-I6iv cells were pre-treated with 1μM DEX or DMSO control for 24 h, and then equally plated onto FN-coated coverslips for 90 mins in the presence of 1μM DEX or DMSO. Scale Bars: 10μm. (h) Adhesion assays of SKOV-I6iv cells pre-treated with 1μM DEX or DMSO control for 24 h, and then equally plated onto FN-coated plates for 1 h in the presence of 1μM DEX or DMSO. Percentage of adhered cells was counted. Data represent means ± SD (n = 3). *$p < 0.05$; **$p < 0.01$; ***$p < 0.001$.

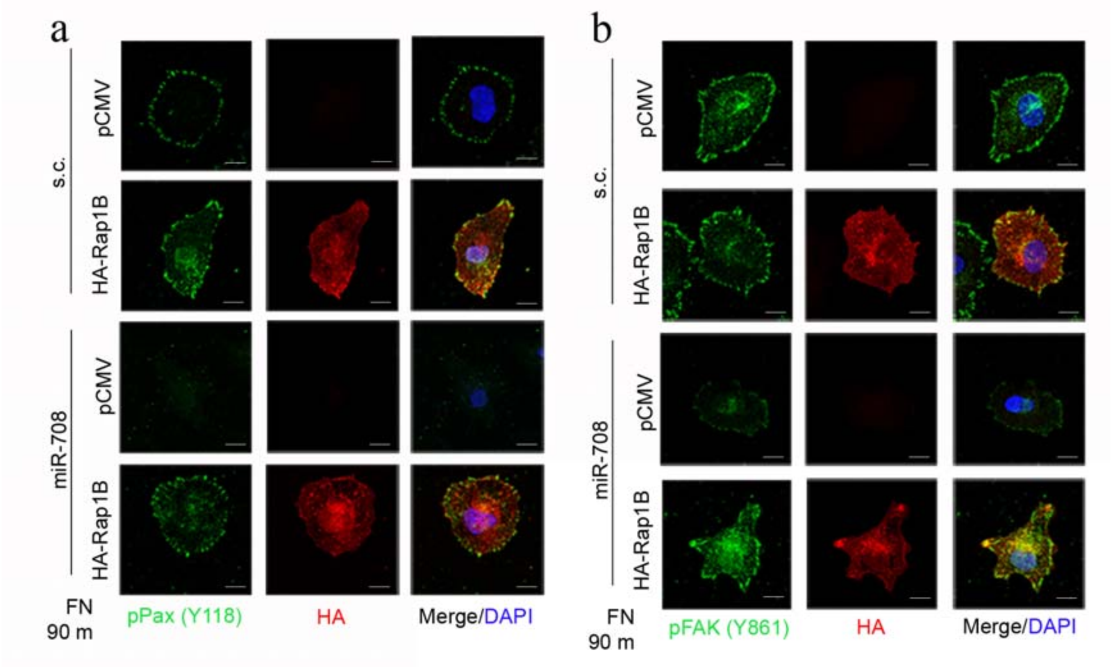

Supplementary Figure 7. MicroRNA-708 mediated destruction of FA formation and cell spreading is rescued by Rap1B expression. (a, b) Confocal microscopy of p-Paxillin (Y118) (a; Green), or p-FAK (Y861) (b; Green), together with HA-tagged-Rap1B (Red), and DAPI (Blue) nuclear staining. SKOV-I6iv cells with indicated transfections were incubated on FN-coated coverslips for 90 mins. Scale Bars: 10μm.

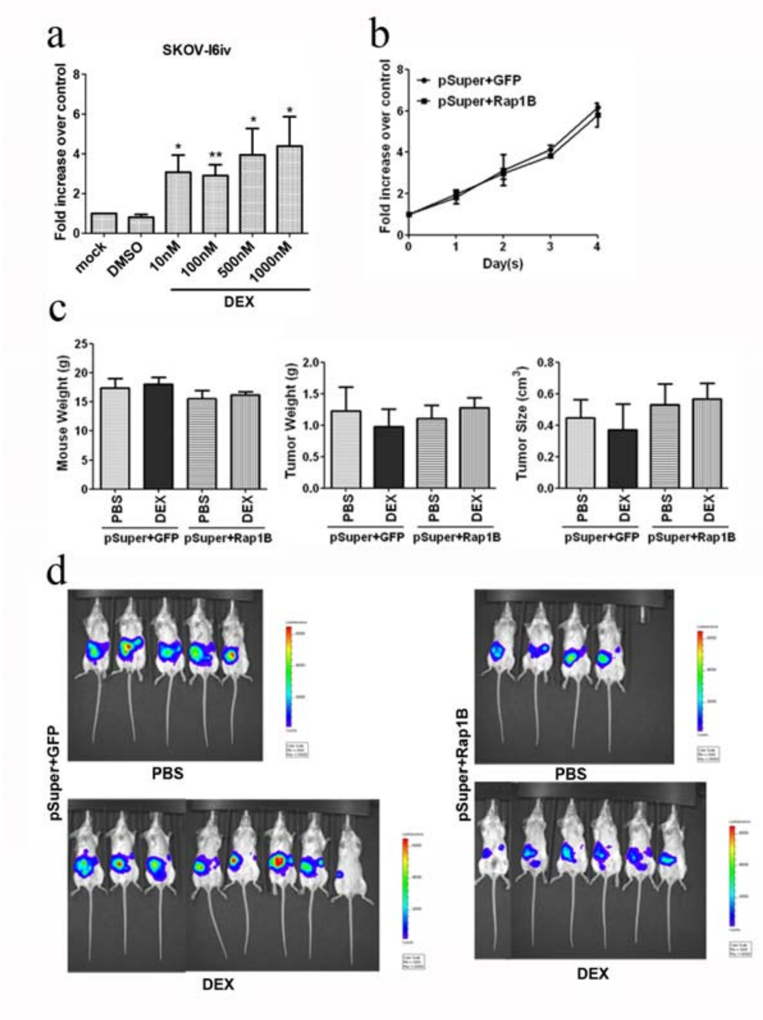

Supplementary Figure 8. Expression of Rap1B reverts glucocorticoid-mediated inhibition of ovarian cancer metastasis *in vivo*. (a) Expression of miR-708 in SKOV-I6iv cells 72h after treatments with increasing amount of DEX (10nM-1μM). Numbers represent normalized means ± SD (n = 3). (b) Cell proliferation assay was performed in SKOV-I6iv-pSuper-GFP-Luc cells stably expressing GFP or Rap1B by MTS reagent. $1x10^3$ cells were cultured and assayed for proliferation rates in 96 wells from 0 to 4 days. Data presented as the normalized means ± SD (n=3). (c) 1 x 10^6 SKOV-I6iv cells transfected with pSuper-GFP-Luc plus GFP or Rap1B were orthotopically injected into mouse ovary. The mouse weight (g), primary tumor weight (g), and tumor size (cm^3) were compared. Data are presented as normalized means to PBS-GFP ± SD (n = 4-12 per group; data were combined from two separate experiments). (d) Bioluminescent images of mice bearing SKOV-I6iv tumors with the indicated plasmid expression and treatment at day 28 after implantation.

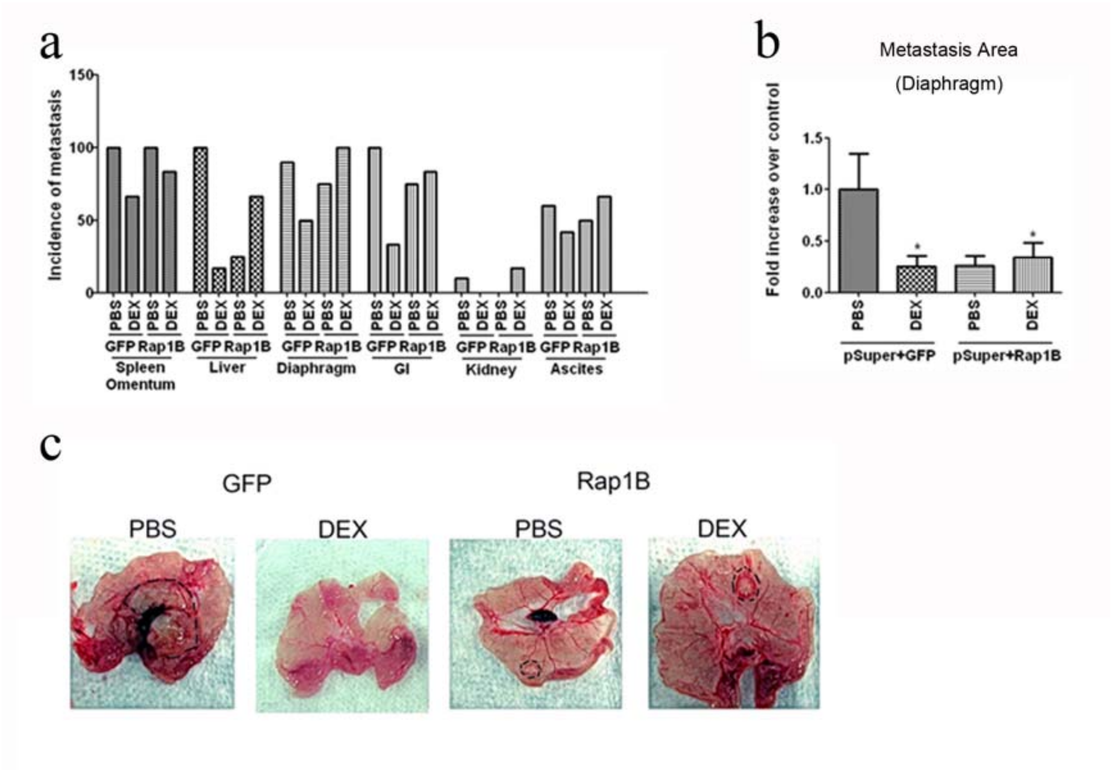

Supplementary Figure 9. Expression of Rap1B reverts glucocorticoid-mediated inhibition of ovarian cancer metastasis *in vivo*. (a) Percentage of tumor incidence in various organs following 30 days of orthotopic implantation of SKOV-I6iv cells transfected with the indicated plasmids and treatments. The tumor incidence was determined by macro metastasis observed from operator's eyes. (b) Quantification results of diaphragm metastases by measuring the area of diaphragm metastatic tumor, and then normalized with total diaphragm area. Data are normalized to the means of PBS-GFP ± SEM (n=4-8). (c) Representative photos of diaphragm in these four groups are shown. Black dashed circles indicate the metastatic tumor area.

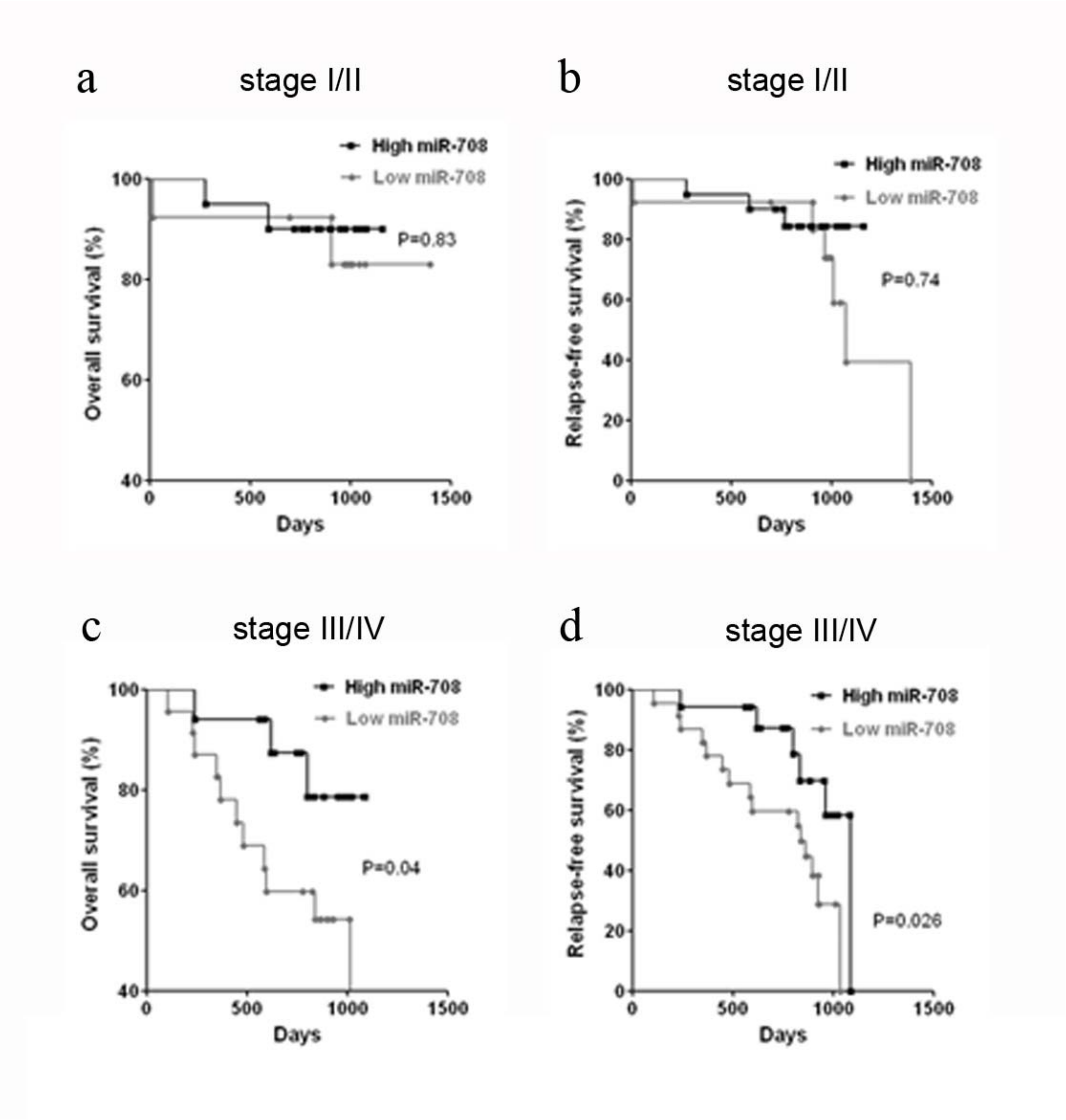

Supplementary Figure 10. Late stage ovarian cancer patients with high miR-708 expressions showed better overall and relapse-free survival. (a, b) Retrospective analysis of Kaplan-Meier plots for miRNA-708 expression in association with overall (a) or relapse-free (b) survival from 33 early stage (stage I/ II) ovarian cancer patients. Patients were split into high and low expression groups based on the median expression of the miR-708. (c, d) Retrospective analysis of Kaplan-Meier plots for miRNA-708 expression in association with overall (c) or relapse-free (d) survival from 40 late stage (stage III/ IV) ovarian cancer patients. Patients were split into high and low expression groups based on the median expression of the miR-708.

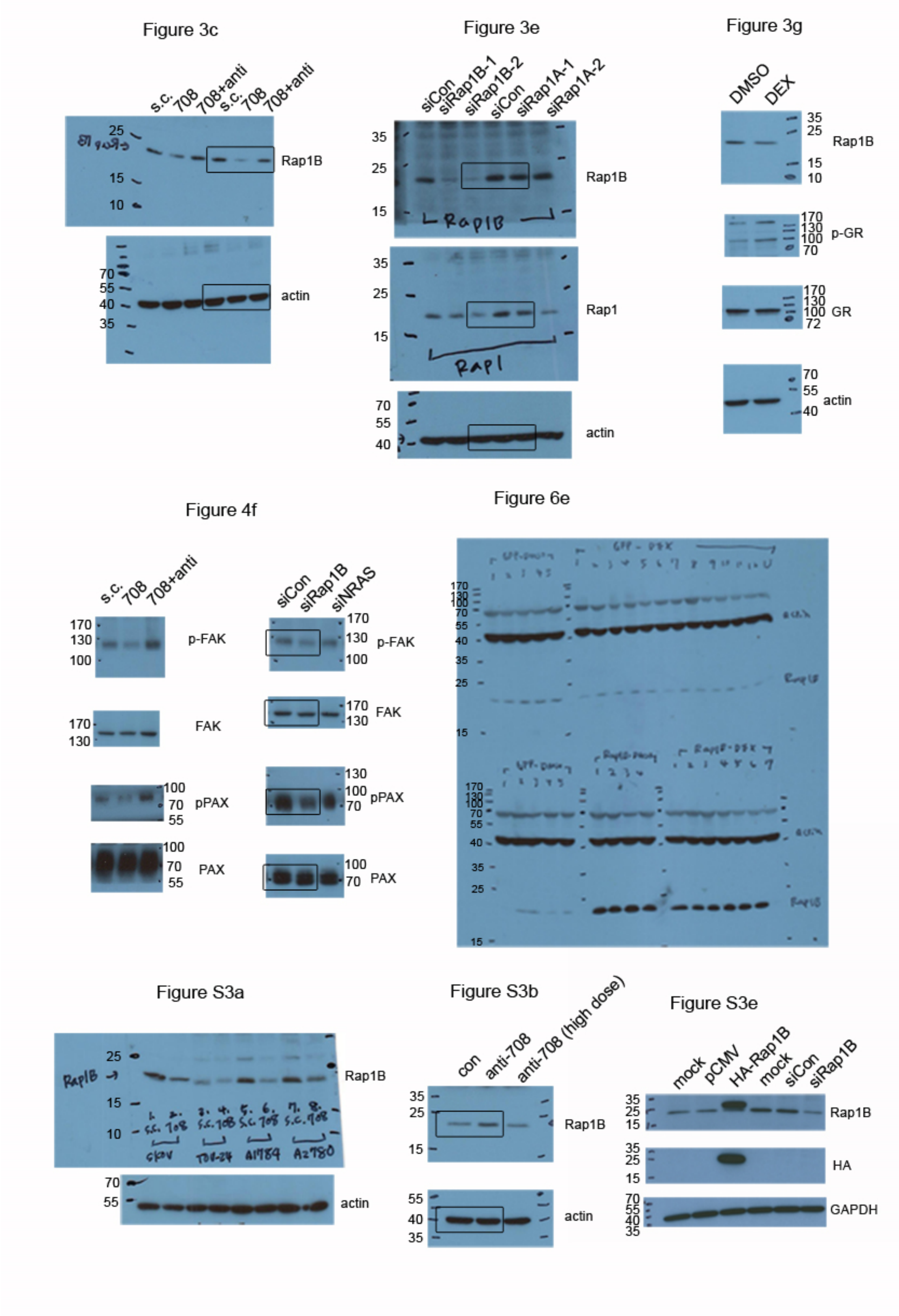

Supplementary Figure 11. Full scans of Western blots

Supplementary Table 1. Primers and siRNAs used in this study

**siRNA sequence**	
Rap1B	AGACACUGAUGAUGUUCCA
GRα	CCGAGAUGUUAGCUGAAAUTT
Rap1A	GUCCACGUUUAACGACUUATT
Odz4	GGAGCUCUAUCUGUUUGAUTT

**qRT-PCR primer sequences (SYBR)**		
	Forward	Reverse
survivin	5'-GACAGAGAAAGAGCCAAGA-3'	5'-CTCCGCAGTTTCCTCAAA-3'
MCAM	5'-ATATCGCTGCTGAGTGAAC-3'	5'-CTGCCTTCCTGTCTCTCA-3'
FN1	5'-AACGACACATTCCACAAG-3'	5'-CTCTGAATCCTGGCATTG-3'
ZEB2	5'-CAAGGATTTCAGGGAGAA-3'	5'-GTCGGAGTCTGTCATATC-3'
AKT2	5'-ACTGAGGAGATGGAAGTG-3'	5'-CCAAGGAGTTTGAGATAGTC-3'
BMI1	5'-GGAGGAGGTGAATGATAA-3'	5'-GTGCTGGCTGAGTAGATCCA-3'
EYA3	5'- GCACATTATTCTTATCCCATTCA-3'	5'- TTGAGATGCTGGCTACTG-3'
AQP1	5'- TCCTGGCTATTGACTACAC-3'	5'- AGTGGTTGCTGAAGTTGT-3'
SGK1	5'-AAGACACAAGGCAGAAGAA-3'	5'- CATTCCGCTCCGACATAA-3'
FOLR1	5'- AAGCACCACAAGGAAAAG-3'	5'- GTAGAACAGCAGGCATTC-3'
NNAT	5'-GCATTTACTGGGTAGGATTC-3'	5'-ACCTGAACACCTCACTTC-3'
RAP1A	5'-TGTCTCACTGCACCTTCAATGGCAT-3'	5'-ACGCCTCCTGAACCAAGGACCA-3'
RAP1B	5'- ACGTCAAGGCGACATCGCCAA-3'	5'-TACTCTGCGAACCTCTCACGCTGT -3'
GRα	5'-TACCCTGCATGTACGACCAA-3'	5'-TCCTTCCCTCTTGACAATGG-3'
ODZ4	5'-AGGATCTTCCCCTCTGGAAA-3'	5'-AAGGTCCTTCACCACCACAG-3'
Human specific GAPDH	5'-TCCTGGTATGACAACGAAT-3'	5'-GGTCTCTCTCTTCCTCTTG-3'
actin	5'-CGGCATCGTCACCAACTG-3'	5'-TCTCAAACATGATCTGGGTCATCT-3'

Primers for cloning		
	Forward	Reverse
pGL3-Rap1B-708 site	ATATGAATTCGGAGAGTACAATAATG	ATATGGCCGGCCGCTACAAGTATCAAAG
pGL4-miR-708 Promo-1	ATTCTCGAGAAAGCAAAGGAGGTAAAAGGAGAG	ATTAAGCTTTCTGCTCCTCCAATGTAA
pGL4-miR-708 Promo-2	ATTCTCGAGTATTGATGTTCGTCTAGG	ATTAAGCTTCGCAGGAAACCAGGAATA
pGL4-miR-708 Promo-3	ATTCTCGAGGGAATTGATCGCAGAGGAG	ATTAAGCTTTTCGCTGCAAATCTCTCT
pGL4-miR-708 Promo-4	ATTGGTACCGCTTTAGTCCAGATGAGG	ATTGCTAGCTTGAGGGCAGTTACCATTT

**Primers for CHIP assay**	Forward	Reverse
Promo-3	AGAACTGAGGTTTCATTG	CAGGAATAGGTGTAGGTA

**miRNA qRT-PCR primer sequences (Taqman)**	
miR-708-RT	5'- GTTGGCTCTGGTGCAGGGTCCGAGGTATTCGCACCAGAGCCAACCCCAGC - 3'
miR-708 Forward Primer	5'- CGGCGGAAGGAGCTTACAATCTA - 3'
U6-RT	5'- GTTGGCTCTGGTGCAGGGTCCGAGGTATTCGCACCAGAGCCAACAAAAATAT - 3'
U6 Forward Primer	5'- TTCCTCCGCAAGGATGACACGC - 3'
Universal Reverse Primer	5'- GTGCAGGGTCCGAGGT - 3'

Supplementary Table 2. Up-regulated (Red) and down-regulated (Green) miRNAs in the *in-vivo* selected SKOV-I6iv cells, compared to the parental SKOV-3 cells.

MicroRNA	SKOV-I6iv	SKOV-3	Fold
hsa-miR-146a	4.0580993	11.784818	211.8234
hsa-miR-708	3.8587475	9.548628	51.6208
hsa-miR-363	2.9562268	7.873919	30.22546
hsa-miR-218-1*	3.0060272	7.7546005	26.88209
hsa-miR-218	4.0611243	7.905321	14.36212
hsa-miR-218	4.242987	7.658477	10.67001
hsa-miR-375	4.8914886	7.5933876	6.506578
hsa-miR-551b	10.672848	8.17047	5.666184
hsa-miR-34b*	5.30775	7.675066	5.159802
hsa-miR-935	5.3027544	7.5791364	4.844615
hsa-miR-200a*	5.427973	7.5778213	4.437812
hsa-miR-486-5p	9.532578	7.4622574	4.199801
hsa-miR-129-3p	7.3172655	5.34254	3.930535
hsa-miR-34c-5p	5.7647347	7.7077827	3.845172
hsa-miR-200a	11.085315	12.985125	3.73164
hsa-miR-486-3p	10.188471	8.318567	3.655082
hsa-miR-429	8.018552	9.846362	3.549979
hsa-miR-200b*	6.486572	8.237177	3.364997
hsa-miR-200b	11.467321	13.172727	3.261205
hsa-miR-130a	11.950855	13.385582	2.70331
hsa-miR-301b	7.5058446	8.794203	2.442499
hsa-miR-95	5.7886105	7.054681	2.405056
hsa-miR-455-5p	6.057778	7.2652516	2.309329
hsa-miR-598	7.0674553	5.869736	2.293768
hsa-miR-1271	6.6547093	7.8448114	2.281689
hsa-miR-1201	11.095105	12.253851	2.232632
hsa-miR-137	7.611634	6.481431	2.188895
hsa-let-7d*	11.194343	10.101569	2.132837
hsa-miR-7	11.88878	10.807728	2.115578
hsa-miR-23b*	6.950777	8.010541	2.08459
hsa-miR-342-5p	6.66874	7.6923227	2.032962
hsa-miR-130b	10.427349	11.43246	2.007098
hsa-miR-760	6.2168236	7.2219186	2.007076

Supplementary Table 3. Top 100 miR-708 target genes predicted by PicTar, Targetscan, and Miranda. Genes predicted by two or more algorithms are shown. Genes in pink were predicted from three algorithms, in orange were from PicTar and Miranda, in green were from PicTar and Targetscan, in purple were predicted from Targetscan and Miranda, respectively.

PicTar	Targetscan	Miranda
KIAA0355	KIAA0355	KIAA0355
RAP1B	RAP1B	RAP1B
FLJ36031		FLJ36031
IQSEC2	IQSEC2	
SETDB1	SETDB1	
SSRP1	SSRP1	
TXNL1	TXNL1	
	SLC44A5	SLC44A5
	RPRD1A	RPRD1A
	C14orf101	C14orf101
	TMEM200B	TMEM200B
	NFE2L2	NFE2L2
	C16orf52	C16orf52
	GPM6A	GPM6A
	EN2	EN2
	AS3MT	AS3MT
	MTSS1	MTSS1

Supplementary Table 4. Clinical features of the frozen tissues from adjacent non-tumor (N), tumors (T), or N/T pairs of ovarian cancer patients obtained from Taipei Veterans General Hospital (TVGH).

**Age (years)**	**≧65**	**< 65**		
	7	22		
**Menopause**	**Yes**	**No**		
	21	8		
**Normal/Tumor**	**Normal**	**Tumor**	**N/T Paired**	
	5	11	13	
**Stage***	**I**	**II**	**III**	**IV**
	4	5	15	4
**Grade***	**Well**	**Moderate**	**Poor**	
	0	3	17	
**Histology***	**Serous**	**Endometroid**	**Clear Cells**	**Mucinous**
	8	7	3	2

*Some data are not available.

Supplementary Table 5. Clinical features between high and low expression of miR-708 in 82 paraffin-embedded primary ovarian tumor specimens from Taipei Veterans General Hospital (TVGH). Patients are split into high and low expression groups based on the median expression of the miR-708.

	**miR-708**		
	**Low (n=41)**	**High (n=41)**	***p value***
**Age (years)**			
≧65	8	6	0.764[1]
< 65	33	35	
**Menopause**	18	20	
**Stage***			
I	6	12	0.201[1]
II	6	7	
III	18	18	
IV	5	1	
**Grade***			
Well	3	0	0.062[1]
Moderate	15	23	
Poor	17	12	
**Histology**			
Serous	17	12	0.451[1]
Endometroid	9	16	
Clear Cell	10	7	
Mucinous	4	4	
Others	1	2	

[1]Fisher's exact test.

*Some data are not available.
